# Multidimensional analysis of the host response reveals prognostic and pathogen-driven immune subtypes among adults with sepsis in Uganda

**DOI:** 10.1186/s13054-022-03907-3

**Published:** 2022-02-08

**Authors:** Matthew J. Cummings, Barnabas Bakamutumaho, Adam Price, Nicholas Owor, John Kayiwa, Joyce Namulondo, Timothy Byaruhanga, Moses Muwanga, Christopher Nsereko, Stephen Sameroff, Rafal Tokarz, Wai Wong, Shivang S. Shah, Michelle H. Larsen, W. Ian Lipkin, Julius J. Lutwama, Max R. O’Donnell

**Affiliations:** 1 Division of Pulmonary, Allergy, and Critical Care Medicine, Department of Medicine, Columbia University Vagelos College of Physicians and Surgeons, 622 West 168th St, PH 8E-101, New York, NY 10032 USA; 2 Center for Infection and Immunity, Mailman School of Public Health, Columbia University, New York, NY USA; 3 Department of Arbovirology, Emerging and Re-Emerging Infectious Diseases, Uganda Virus Research Institute, Entebbe, Uganda; 4 Immunizable Diseases Unit, Uganda Virus Research Institute, Entebbe, Uganda; 5 Entebbe General Referral Hospital, Ministry of Health, Entebbe, Uganda; 6 Division of Infectious Diseases, Department of Pediatrics, Vagelos College of Physicians and Surgeons, Columbia University, New York, NY USA; 7 Department of Microbiology and Immunology, Albert Einstein College of Medicine, Bronx, NY USA; 8 Department of Pathology and Cell Biology, Vagelos College of Physicians and Surgeons, Columbia University, New York, NY USA; 9 Department of Epidemiology, Mailman School of Public Health, Columbia University, New York, NY USA

**Keywords:** Sepsis, Biomarkers, Tuberculosis, High-throughput nucleotide sequencing, Uganda, Africa

## Abstract

**Background:**

The global burden of sepsis is concentrated in sub-Saharan Africa, where severe infections disproportionately affect young, HIV-infected adults and high-burden pathogens are unique. In this context, poor understanding of sepsis immunopathology represents a crucial barrier to development of locally-effective treatment strategies. We sought to determine inter-individual immunologic heterogeneity among adults hospitalized with sepsis in a sub-Saharan African setting, and characterize associations between immune subtypes, infecting pathogens, and clinical outcomes.

**Methods:**

Among a prospective observational cohort of 288 adults hospitalized with suspected sepsis in Uganda, we applied machine learning methods to 14 soluble host immune mediators, reflective of key domains of sepsis immunopathology (innate and adaptive immune activation, endothelial dysfunction, fibrinolysis), to identify immune subtypes in randomly-split discovery (N = 201) and internal validation (N = 87) sub-cohorts. In parallel, we applied similar methods to whole-blood RNA-sequencing data from a consecutive subset of patients (N = 128) to identify transcriptional subtypes, which we characterized using biological pathway and immune cell-type deconvolution analyses.

**Results:**

Unsupervised clustering consistently identified two immune subtypes defined by differential activation of pro-inflammatory innate and adaptive immune pathways, with transcriptional evidence of concomitant CD56(-)/CD16( +) NK-cell expansion, T-cell exhaustion, and oxidative-stress and hypoxia-induced metabolic and cell-cycle reprogramming in the hyperinflammatory subtype. Immune subtypes defined by greater pro-inflammatory immune activation, T-cell exhaustion, and metabolic reprogramming were consistently associated with a high-prevalence of severe and often disseminated HIV-associated tuberculosis, as well as more extensive organ dysfunction, worse functional outcomes, and higher 30-day mortality.

**Conclusions:**

Our results highlight unique host- and pathogen-driven features of sepsis immunopathology in sub-Saharan Africa, including the importance of severe HIV-associated tuberculosis, and reinforce the need to develop more biologically-informed treatment strategies in the region, particularly those incorporating immunomodulation.

**Supplementary Information:**

The online version contains supplementary material available at 10.1186/s13054-022-03907-3.

## Introduction

The global burden of sepsis is concentrated in sub-Saharan Africa (SSA), where nearly 40% of all sepsis cases occur and up to 65% of all deaths are sepsis-related [1, 2]. In this context, where epidemic HIV, extensive pathogen diversity, and limited critical care capacity challenge effective management of life-threatening infections, clinical trials of sepsis treatment protocols developed in high-income countries (HICs) have repeatedly shown harm [3, 4]. As treatment efficacy likely depends, in part, on modifying complex host responses incited by an array of pathogens, imprecise understanding of biological heterogeneity inherent to sepsis in SSA represents a crucial barrier to development of locally-effective management strategies [5].

In high-income countries (HICs), where sepsis typically affects older adults with severe bacterial infections [2], comprehensive profiling of the host response has established immunopathological models defined by dynamic features of inflammation, dysregulated endothelial and cellular metabolic function, and immunosuppression. Although identification of effective, biologically-driven therapeutics for sepsis in HICs remains elusive, such models have laid the conceptual groundwork for development of more precise treatment strategies [5–10]. In SSA, where sepsis disproportionately affects young, HIV-infected adults and high-burden pathogens are unique [3], data informing locally-relevant models of sepsis immunopathology are scarce.

Among a prospective cohort of adults hospitalized with suspected sepsis in Uganda [11], we applied a multidimensional approach to determine inter-individual immunologic heterogeneity and identify distinct, biologically-driven host response subtypes that may have prognostic and therapeutic relevance in the unique SSA context.

## Methods

### Study setting, participants, and design

In this exploratory study, we analyzed data and blood samples from a prospective observational cohort (Research in the Epidemiology of Severe and Emerging Infections in Uganda; RESERVE-U) of adults (age ≥ 18 years) hospitalized with severe, undifferentiated infection (suspected sepsis) at Entebbe General Referral Hospital (EGRH) in central Uganda from April 2017-August 2019 [11]. EGRH is a 200-bed public district referral hospital with a catchment area of approximately 3 million persons. In the primary catchment area, HIV prevalence is approximately 6% and malaria is endemic [11]. Representative of a general district hospital in SSA, there is no intensive care unit at EGRH. No vasopressor or inotropic agents are available and intravenous (IV) fluid is typically delivered as 250–500 ml infusions of crystalloid. As no piped oxygen was available at EGRH during the enrollment period, oxygen concentrators were provided to hospital wards as part of the study program.

Patients were included in the parent RESERVE-U study if they fulfilled the following criteria: (1) age ≥ 18 years, (2) reported a history of fever or had a recorded axillary temperature of ≥ 37.5ºC at presentation, (3) had clinical illness severe enough to warrant admission to hospital, and (4) were able to provide informed consent or had a surrogate available to do so. Patients were excluded if they presented following trauma or were admitted to a non-medical ward. Study enrollment occurred within 24 h of hospital admission. Further details of the RESERVE-U study have been published and are summarized in the supplement [11].

Among patients enrolled in the parent RESERVE-U study, we applied a multidimensional approach to dissect the host immune response (Additional file 1: Figure E1). First, given the unsupervised nature of our analyses, we randomly split our parent cohort of 288 patients into discovery (70%; N = 201) and internal validation (30%; N = 87) sub-cohorts. We then performed unsupervised clustering on 14 soluble host mediators, chosen a priori to reflect putative domains of sepsis immunopathology (innate and adaptive immune activation, endothelial dysfunction, fibrinolysis), to identify immune subtypes in the discovery and internal validation cohorts independently. Molecular signatures defining each immune subtype in the discovery cohort were explored using classification, regression, and network analyses. In parallel, we performed unsupervised clustering on whole-blood RNA-sequencing data from a consecutive subset of 128 patients in the parent cohort to identify transcriptional subtypes, which we characterized using biological pathway and immune cell-type deconvolution analyses. We compared microbiological characteristics and clinical outcomes across soluble mediator- and transcriptionally-derived immune subtypes, and integrated these results to harmonize the biological and clinical relevance of our findings.

### Pathogen diagnostics

For all enrolled patients in the RESERVE-U study, rapid testing was performed for malaria, influenza, and HIV; for HIV-infected patients testing for tuberculosis (TB) was also performed. Testing for these pathogens was informed by World Health Organization (WHO) guidelines for management of sepsis and septic shock in resource-limited hospitals in sub-Saharan Africa (WHO Integrated Management of Adolescent and Adult Illness [IMAI] District Clinician Manual) [12]. The WHO IMAI guidelines emphasize rapid testing for malaria and HIV, a low threshold for TB testing among HIV-infected patients, and consideration of testing or empiric treatment for influenza. Further details are in the supplement.

### Serum immunoassays

From cryopreserved serum samples collected at the time of study enrollment, interleukin (IL)-6, IL-8, IL-10, interferon (IFN)-γ, IFN-γ-induced protein-10/C-X-C motif chemokine 10 (IP-10/CXCL10), macrophage inflammatory protein-1-alpha/chemokine (C–C motif) ligand 3 (MIP-1α/CCL3), macrophage inflammatory protein-1-beta/chemokine (C–C motif) ligand 4 (MIP-1β/CCL4), tumor necrosis factor-alpha (TNF-α), angiopoietin-1 (Ang-1), angiopoietin-2 (Ang-2), macrophage migration inhibitory factor (MIF), plasminogen activator inhibitor-1 (PAI-1), soluble TNF-receptor type 1 (sTNFR1), and soluble IL-2 receptor alpha/soluble CD25 (sIL-2RA/sCD25), were quantified using custom Luminex 200 system kits (Luminex, Austin, TX, USA) from MilliporeSigma (Burlington, Massachusetts, USA) and R&D Systems (Minneapolis, MN, USA). Further details are in the supplement.

### Whole-blood RNA isolation, library preparation, and sequencing

From cryopreserved whole-blood samples collected in PAXgene blood RNA tubes (PreAnalytiX, Qiagen/BD, Hombrechtikon, Switzerland) at the time of study enrollment, RNA was isolated and purified using PAXgene blood RNA kits (Qiagen, Hilden, Germany). RNA sequencing libraries were prepared using the NEBNext Ultra RNA Library Prep Kit (NEB, Ipswich, MA, USA). Sequencing libraries were multiplexed and analyzed using a 2 × 150 paired-end configuration on the Illumina HiSeq 4000 platform (Illumina, Inc., San Diego, CA, USA). Adapters were trimmed from raw reads using Trimmomatic and sequencing data quality was assessed with FastQC [13, 14]. Sequencing reads were aligned to the human genome (GRCh38) using STAR and transcript quantification was performed using the R-subread package’s featureCounts utility [15, 16]. Further details are in the supplement.

### Analysis of soluble immune mediators

To identify immune subtypes, we applied unsupervised clustering methods to serum mediator concentrations from patients in the discovery and internal validation cohorts independently. Clustering is a form of unsupervised machine learning that seeks to classify individual data points into clusters, or groups, based on metrics of similarity (e.g., distance, correlation), when the underlying group structure is unknown [17]. Specifically, we applied agglomerative hierarchical clustering (using Ward’s method and Euclidean distance) to log_10_ transformed, scaled, and centered mediator concentrations to initiate a cluster partition, followed by a k-means procedure to consolidate cluster membership [18]. We used within-cluster-sum-of-squares to determine the optimal number of clusters, which we confirmed using over 20 indices of cluster validity and stability [19]. We visualized between-cluster variance in mediator concentrations using principal component analysis and standardized heatmaps. Further details are in the supplement.

To identify the most influential variables driving cluster (i.e. immune subtype) assignment in the discovery cohort, we determined representation of mediator variables on the first two principal components by calculating squared factor loadings for each variable. Separately, we applied a gradient-boosted decision tree algorithm, trained to predict cluster assignment, to our mediator variables, and identified the most important discriminatory variables using their respective split gain values. Next, to explore longitudinal, cluster-specific changes in soluble mediators over the course of illness, we evaluated the relationship between mediator concentrations and reported duration of illness at admission (obtained via patient or surrogate) using robust regression, with patient-level datapoints stratified by cluster assignment. Lastly, to determine inter-mediator relationships within each discovery cohort cluster and identify “central” mediators around which each cluster may be coordinated, we constructed force-directed weighted correlation networks [20–22]. Further details are in the supplement.

### Analysis of whole-blood RNA-sequencing data

Independent of soluble mediator analyses and using the same parameters, we performed unsupervised hierarchical clustering of whole-blood RNA-sequencing data from a consecutively enrolled subset of patients (N = 128) in the RESERVE-U cohort. Following determination of the optimal number of clusters (i.e. transcriptional subtypes) using within-cluster-sum-of-squares, we identified genes that were differentially expressed across each cluster based on a log-fold change ≥|1| and Benjamini-Hochberg-adjusted p-value ≤ 0.01 [23, 24]. Differentially expressed gene sets were selected for biological pathway analysis (Ingenuity Pathway Analysis, Qiagen), results of which were examined to infer functional differences between identified clusters. Using the ImmQuant software package and IRIS and DMAP mRNA compendiums, we applied digital cell quantification (DCQ) deconvolution to our differentially expressed gene sets to infer relative immune cell quantities across transcriptional clusters [25–27]. Further details are in the supplement.

### Statistical analyses

In presentations of clinical data, continuous variables are expressed as medians (interquartile range [IQR]) and categorical variables are summarized as counts and percentages with 95% confidence intervals (CI) and two-sided p-values presented where relevant. Clinical and microbiological characteristics across immune mediator- and transcriptional subtypes were compared using Chi-squared, Fisher exact, or Wilcoxon rank-sum tests as appropriate. Univariable and multivariable logistic regression were used to compare the primary outcome of 30-day mortality across identified immune subtypes. Given the exploratory nature of this study, no adjustment for multiple comparisons was performed unless indicated. For the primary outcome of 30-day vital status, mortality was not imputed if vital status was unknown, and a sensitivity analysis was performed to account for potential bias due to loss-to-follow-up. Further details are in the supplement.

Analyses were performed using R (v3.6.1, R Foundation for Statistical Computing, Vienna, Austria) via the RStudio (v1.4.1106) environment, with specific packages detailed in the supplement. Biological pathway and immune deconvolution analyses of RNA-sequencing data were performed using Ingenuity Pathway Analysis (Qiagen, Hilden, Germany) and ImmQuant, respectively [25]. Study overview and flow diagrams were created with Biorender.

## Results

### Participants

Of 301 adult patients enrolled in the RESERVE-U cohort, 288 (96%) and 128 (43%) had serum and whole-blood RNA samples available for analysis, respectively, and were included in this study (Additional file 1: Figure E2). Characteristics of the 288 patients for whom soluble mediators were analyzed, nearly 90% of whom had ≥ 1 quick-Sepsis-related Organ Failure Assessment (qSOFA) criterion, are presented in Table 1. Characteristics of patients who had RNA samples analyzed were similar to those who did not (Additional file 1: Table E1).

**Table 1:** Patient characteristics stratified by discovery and internal validation cohorts

Patient characteristic	All patients (N = 288)	Discovery cohort (N = 201)	Internal validation cohort (N = 87)
Female sex, n (%)	171/288 (59.4)	116/201 (57.7)	55/87 (63.2)
Age, years, median [IQR]	32 [26, 42]	32 [26, 40]	32 [27, 43]
Duration of illness prior to admission, days, median [IQR]^a^	4 [3, 7]	4 [3, 7]	4 [3, 7]
History of fever, n (%)	288/288 (100.0)	201/201 (100.0)	87/87 (100.0)
Night sweats	225/288 (78.1)	161/201 (80.1)	64/87 (73.6)
Headache	227/288 (78.8)	158/201 (78.6)	69/87 (79.3)
Cough	178/288 (61.8)	125/201 (62.2)	53/87 (60.9)
Diarrhea	100/288 (34.7)	70/201 (34.8)	30/87 (34.5)
Shortness of breath	66/288 (22.9)	47/201 (23.4)	19/87 (21.8)
Dysuria	39/288 (13.5)	31/201 (15.4)	8/87 (9.2)
Received antibiotic or antimalarial agent prior to admission, n (%)	102/288 (35.4)	70/201 (34.8)	32/87 (36.8)
Temperature ≥ 38 °C, n (%)	104/288 (36.1)	76/201 (37.8)	28/87 (32.2)
Temperature < 36 °C, n (%)	84/288 (29.2)	58/201 (28.9)	26/87 (29.9)
Heart rate, beats/min, median [IQR]	98 [87,109]	98 [86,108]	98 [90,111]
Respiratory rate, beats/min, median [IQR]	22 [21, 26]	22 [21, 26]	22 [20, 26]
Systolic blood pressure, mmHg, median [IQR]	103 [91,117]	104 [91,118]	100 [92,113]
Oxygen saturation, %, median [IQR]	97 [95,98]	97 [96,98]	97 [95,98]
Encephalopathy, n (%)^b^	57/288 (19.8)	40/201 (19.9)	17/87 (19.5)
qSOFA score ≥ 2, n (%)^c^	129/288 (44.8)	88/201 (43.8)	41/87 (47.1)
qSOFA score ≥ 1, n (%)^c^	253/288 (87.8)	174/201 (86.6)	79/87 (90.8)
Modified SIRS score ≥ 2, n (%)^d^	247/288 (85.8)	173/201 (86.1)	74/87 (85.1)
MEWS, median [IQR]	3 [2, 5]	3 [2, 4]	3 [2, 5]
UVA score, median [IQ R]	3 [2, 4]	3 [1, 4]	2 [2, 4]
Shock, n (%)^e^	41/288 (14.2)	28/201 (13.9)	13/87 (14.9)
Acute respiratory failure, n (%)^f^	61/288 (21.2)	39/201 (19.4)	22/87 (25.3)
Severe anemia, n (%)^g^	56/288 (19.4)	39/201 (19.4)	17/87 (19.5)
HIV-infected, n (%)	154/286 (53.8)	106/199 (53.2)	48/87 (55.2)
WHO clinical stage 3 or 4, n (%)	125/154 (81.2)	91/106 (85.8)	34/48 (71.0)
Newly diagnosed HIV-infection, n (%)	20/154 (13.0)	12/106 (11.3)	8/48 (16.7)
On ART prior to admission, n (%)^h^	91/134 (67.9)	63/94 (67.0)	28/40 (70.0)
On TMP-SMX prior to admission, n (%)^h^	94/134 (70.1)	65/94 (69.1)	29/40 (72.5)
Malaria RDT positive, n (%)	59/283 (20.8)	38/197 (19.3)	21/86 (24.4)
Microbiological TB positive, n (%)^i^	51/288 (17.7)	35/201 (17.4)	16/87 (18.4)
Urine TB-LAM positive	40/122 (32.8)	27/83 (32.5)	13/39 (33.3)
Influenza PCR positive, n (%)	17/262 (6.5)	14/184 (7.6)	3/78 (3.8)
Death in-hospital or transfer, n (%)	40/288 (13.9)	28/201 (13.9)	12/87 (13.8)
Duration of hospitalization, days, median [IQR]^j^	5 [3, 8]	5 [3, 7]	5 [3, 8]
KPS ≤ 70 at alive discharge, n (%)	20/246 (8.1)	12/173 (6.9)	8/73 (11.0)
Death at 30-days post-discharge, n (%)	62/260 (23.8)	44/179 (24.6)	18/81 (22.2)

IQR: interquartile range, qSOFA: quick sequential (sepsis-related) organ failure assessment, SIRS: systemic inflammatory response syndrome, MEWS: modified early warning score, UVA: universal vital assessment, HIV: human immunodeficiency virus, WHO: World Health Organization, ART: anti-retroviral therapy, RDT: rapid diagnostic test, TB: tuberculosis, LAM: lipoarabinomannan, PCR: polymerase chain reaction

^a^Unknown for 1 patient

^b^Anything other than “Alert” on AVPU (alert, responsive to voice, responsive to pain, unresponsive) mental status assessment

^c^Systolic blood pressure ≤ 100 mmHg, respiratory rate ≥ 22 breaths/min, and encephalopathy, latter defined using AVPU scale

^d^Temperature ≥ 38 °C or < 36 °C, heart rate ≥ 90 beats/min, respiratory rate ≥ 20 breaths/min

^e^Systolic blood pressure ≤ 90 mmHg despite administration of ≥ 1 L of intravenous fluid

^f^Oxygen saturation ≤ 90% or respiratory rate ≥ 30 breaths/min

^g^Hemoglobin < 9 g/dl or administration of blood transfusion

^h^Denominator is number with known HIV-infection prior to admission

^i^Positive result by sputum Xpert Ultra or smear or urine TB-LAM

^j^Unknown for 11 patients

### Unsupervised clustering of soluble mediators consistently reveals two immune subtypes among adults with suspected sepsis in Uganda

**Fig. 1:**
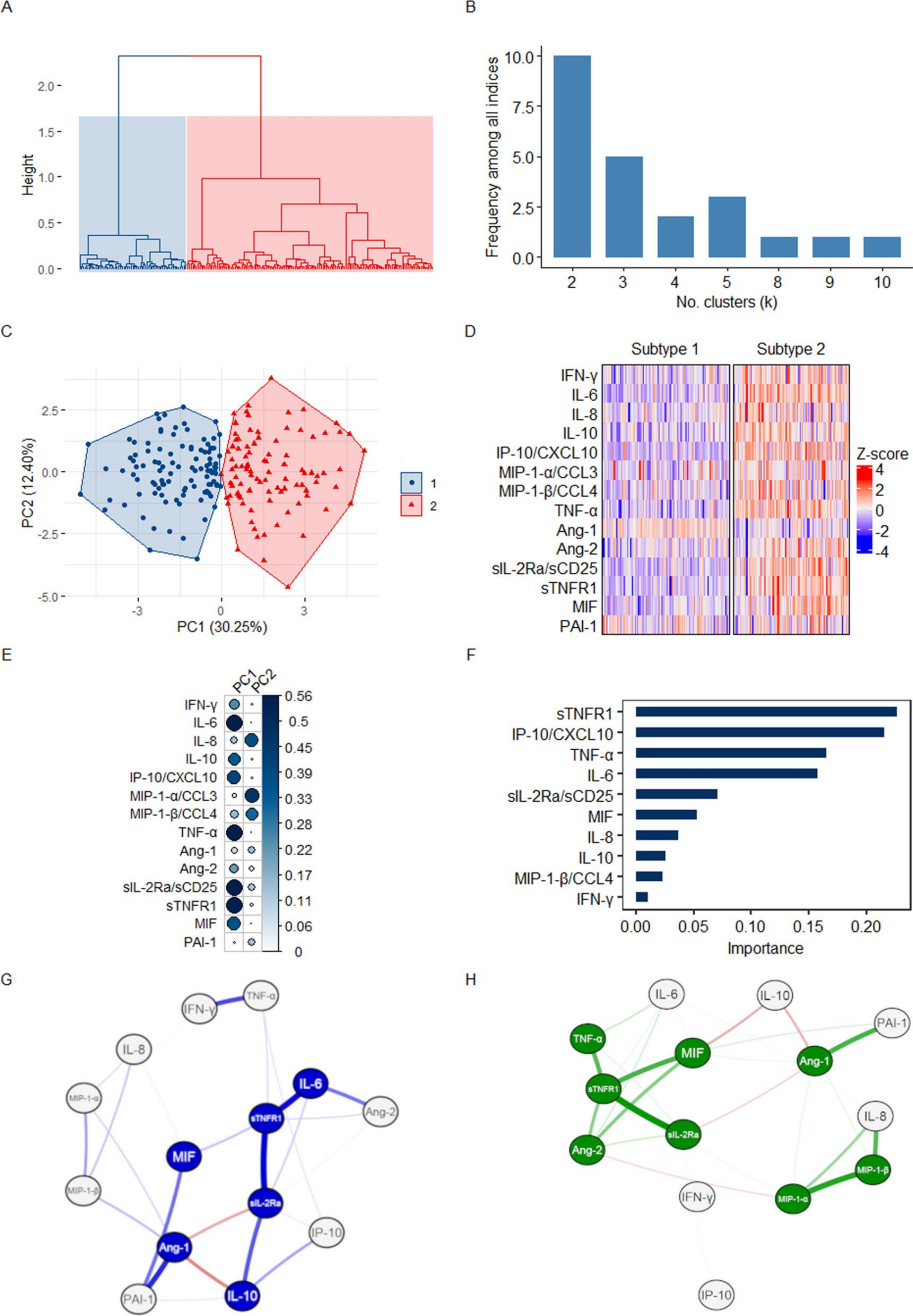
Soluble mediator-derived immune subtypes in discovery cohort. **a** Unsupervised hierarchical clustering of 14 serum mediators reflecting innate and adaptive immune activation, endothelial dysfunction, and fibrinolysis; dendrogram indicates cluster partition prior to k-means consolidation (N = 201). **b** Optimal cluster partitions suggested by cluster stability and validation indices as per NbClust package. **c** First two principal components plotted with the proportion of variance explained by each component; individuals stratified by cluster (subtype) assignment (N = 201). **d** Heatmap of z-score standardized soluble mediator concentrations, stratified by cluster (subtype) (N = 201). **e** Squared factor loadings for all serum mediators across the first two principal components in the discovery cohort; higher loading value indicates greater importance for each variable in explaining variance across each principal component (N = 201). **f** Importance of serum mediator variables in construction of gradient-boosted decision tree algorithm designed to predict cluster (subtype) assignment in discovery cohort (N = 201); 10 most important variables presented. Force-directed correlation networks based on the Fruchterman-Reingold method in discovery cohort subtype 1 **g** and subtype 2 **h**; each mediator variable was set as a network node with between-mediator correlations significant at p-value ≤ 0.05 indicated by weighted edges (blue and green edges indicate positive correlation, red edges indicate negative correlation, edge width indicates strength of correlation) (N = 201). Nodes with blue and green shading indicate those mediators considered central in the subtype 1 and 2 networks, respectively, defined as those with ≥ 1 centrality metric (strength, closeness, or betweenness) above the standardized cluster mean (z-score > 0)

In the discovery and internal validation cohorts, a two cluster (i.e. two subtype) model was determined to be optimal based on multiple indices of cluster stability and validity (Fig. 1a, b and Additional file 1: Figure E3A-E3B, Tables E2-E3), with clear between-cluster separation across the first principal component of mediator variance (Figs. 1c and Additional file 1: Figure E3C). In both the discovery and internal validation cohorts, soluble mediator subtype 2 (S2) was associated with significantly higher concentrations of key mediators promoting inflammation (MIF, IFN-γ, IL-6, TNF-α), T-cell activation and tolerance (sIL-2Ra/sCD25), and neutrophil (IL-8), monocyte/macrophage, NK, Th1, and dendritic-cell (IP-10/CXCL10, MIP-1α/CCL3, MIP-1β/CCL4) chemotaxis (Figs. 1d, Additional file 1: Figure E3D, E4-E5, Tables E4-E5). Although these markers were observed alongside elevated concentrations of anti-inflammatory mediators (sTNFR1 and IL-10), ratios of IL-6/IL-10, IFN-γ/IL-10, and TNF-α/sTNFR1 were consistently higher in subtype 2, suggesting imbalance towards a more pro-inflammatory state (Additional file 1: Figures E4-E5, Tables E4-E5). While concentrations of Ang-1, which promotes endothelial stability and dampening of inflammation, were higher in subtype 1 (S1), those of Ang-2, a pro-inflammatory mediator of endothelial activation and destabilization, were significantly higher in S2, as was the ratio of Ang-2/Ang-1. Examination of squared factor loadings and gradient-boosted decision tree models both identified sTNFR1, IP-10/CXCL10, TNF-α, IL-6, and sIL-2Ra/sCD25 as the most influential mediators driving subtype assignment (Fig. 1e, f).

### Network analyses demonstrate subtype-specific responses coordinated around shared and distinct mediators

Force-directed network structures were unique to each discovery cohort subtype and were differentially coordinated around a core of central mediators. Although some mediators were central in both subtypes, the core S1 network included more anti-inflammatory and endothelial-protective mediators (sTNFR1, IL-10, Ang-1) (Fig. 1g). In contrast, S2 was coordinated around more pro-inflammatory and chemotactic (MIP-1α/CCL3, MIP-1β/CCL4, TNF-α) and endothelial-destabilizing (Ang-2) mediators (Fig. 1h).

### Profiles of divergent immune activation and endothelial dysfunction distinguish host subtypes throughout the course of illness

**Fig. 2:**
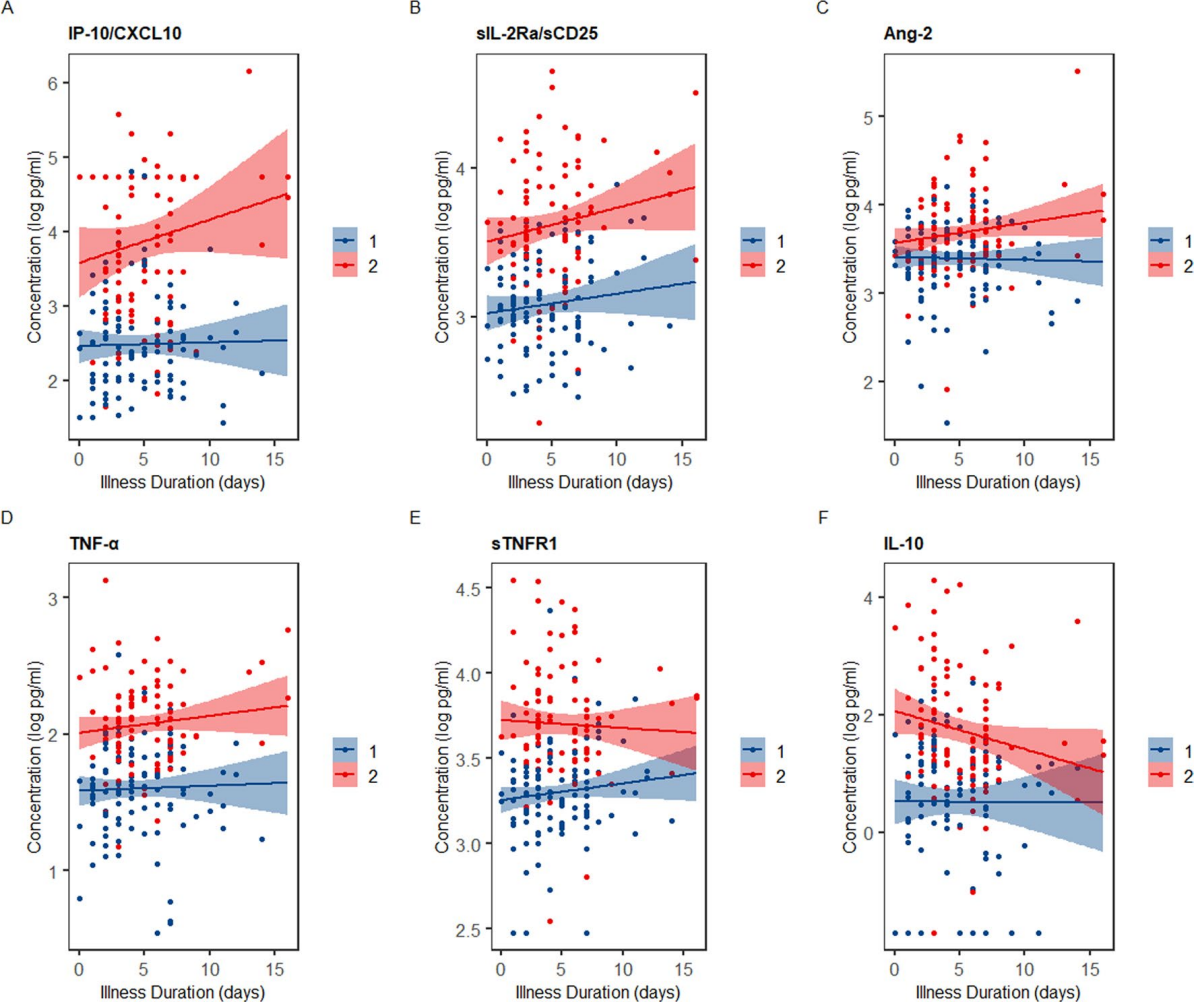
Serum mediator concentrations over the reported course of illness in the discovery cohort, stratified by immune subtype. **a–f** Concentrations of soluble mediators over the reported course of illness, with robust regression lines and 95% confidence intervals, stratified by immune subtype (N = 198; 1 patient with unknown illness duration, 2 patients with extreme outliers in illness duration excluded). For example, an individual data point corresponding to “day 0” represents the serum mediator concentration for a patient who was admitted to hospital on the day of illness onset, while that corresponding to “day 5” represents a patient who was admitted to hospital on day 5 of illness

Nearly all mediators were generally higher in S2 throughout the reported course of illness, with the exception of Ang-1, which appeared consistently higher in S1 (Fig. 2, Additional file 1: Figure E6). While concentrations of many mediators remained stably divergent, there were disproportionate decreases over time for IL-10 and sTNFR1 in S2. In contrast, concentrations of IP-10/CXCL10, sIL2-Ra/sCD25, and Ang-2 disproportionately increased over time in S2.

### Immune subtypes are associated with differential profiles of physiologic derangement, organ dysfunction, and mortality

**Fig. 3:**
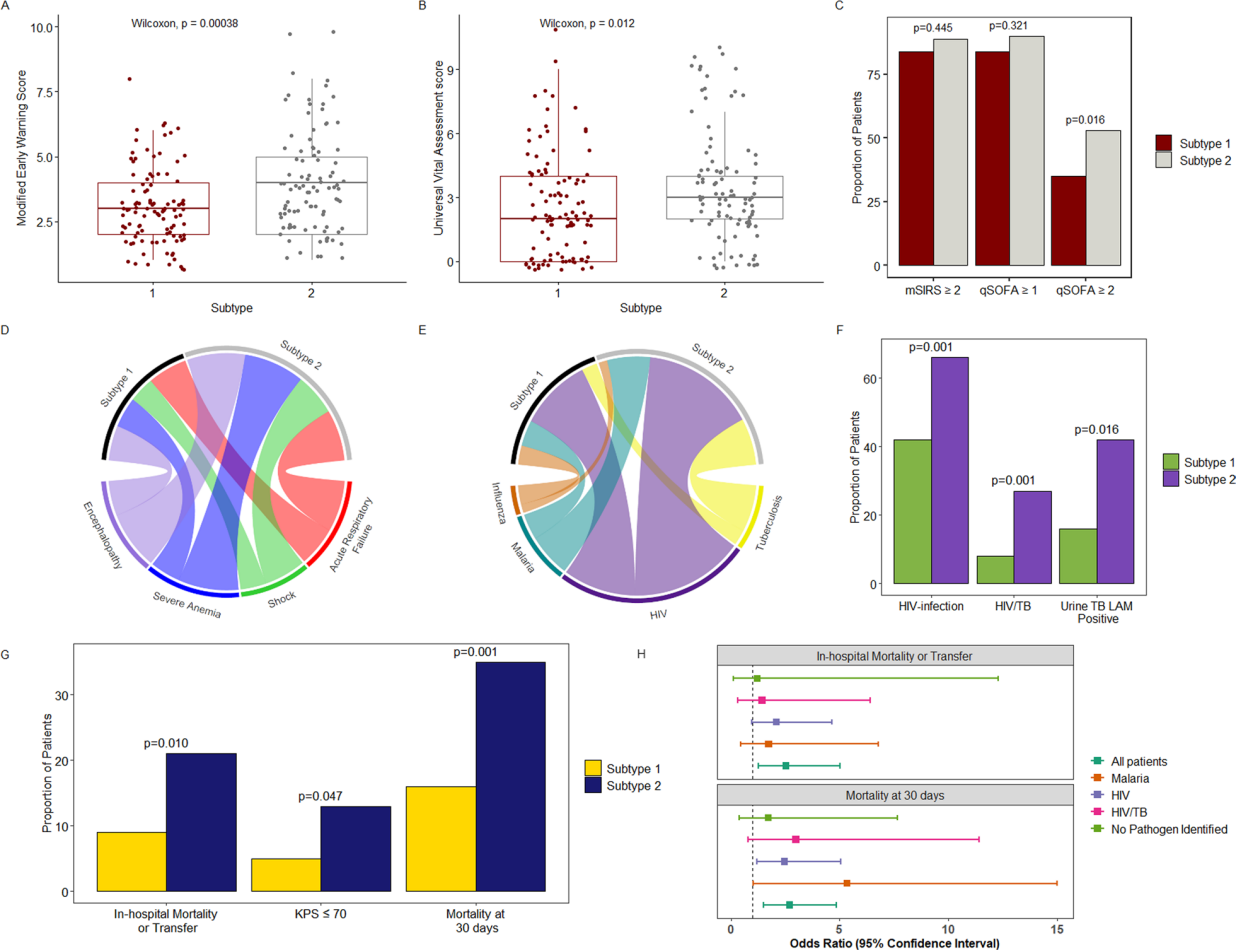
Illness severity scores, distributions of organ failures and pathogens, and outcomes stratified by immune subtypes. **a–c** Modified Early Warning Score, Universal Vital Assessment score, modified systemic inflammatory response syndrome [mSIRS], and quick Sepsis-related Organ Failure assessment [qSOFA] scores stratified by immune subtype in the discovery cohort; p-values in 3C represent Chi-squared test with continuity correction (N = 201). **d** Chord plot indicating proportion of patients with specific organ failures across each subtype in the discovery cohort; a wider chord band indicates a greater proportion of patients with each corresponding organ failure (N = 201, proportions in subtype 2 vs. 1 as follows: shock: 17.7% vs. 10.5%; acute respiratory failure: 21.9% vs. 17.1%; severe anemia: 26.0% vs. 13.3%; encephalopathy: 25.0% vs. 15.2%). **e** Chord plot indicating proportion of patients with specific infections across each subtype in the discovery cohort; a wider chord band indicates a greater proportion of patients with each corresponding infection (N = 201, proportions in subtype 2 vs. 1 as follows: HIV: 65.6% vs. 42.0%, tuberculosis: 27.1% vs. 8.6%, malaria: 24.5% vs. 14.6%, influenza: 4.5% vs. 10.5%). **f** Proportions of patients with known HIV-infection status (N = 199), HIV-associated TB (N = 199), and positive urine TB-LAM results (among those tested, N = 83) across each immune subtype in the discovery cohort. **g** In-hospital outcome (N = 288), impaired functional status [Karnofsky Performance Status; KPS] among hospital survivors (N = 246), and 30-day vital status (N = 260) across each subtype in a pooled cohort of patients from the discovery and internal validation cohorts; p-values in 3F and 3G represent Chi-squared test with continuity correction. **h** Forest plot indicating univariable (unadjusted) odds ratios for in-hospital outcome and 30-day mortality among patients in subtype 2 vs. subtype 1, stratified by key pathogen groups in pooled discovery and internal validation cohort [patients with influenza omitted given small number of events in that pathogen group; for visualization, upper limit of 95% confidence interval for 30-day mortality truncated at 15 for patients with malaria (upper limit 27.57)

In both the discovery and internal validation cohorts, Universal Vital Assessment (UVA) and Modified Early Warning Scores (MEWS), physiologic indices of clinical severity feasible for use in resource-limited settings, were significantly higher among patients in S2, as were proportions of patients with ≥ 2 qSOFA criteria (Fig. 3a-c, Additional file 1: Tables E2-E3). Consistently, patients in S2 also had more extensive organ dysfunction, including higher prevalence of shock, acute respiratory failure, severe anemia, and encephalopathy (Fig. 3d, Additional file 1: Tables E2-E3).

Given consistent biological and clinical findings in the discovery and internal validation cohorts, we analyzed in-hospital and 30-day outcomes in a pooled sample of patients from both cohorts. In this analysis, mortality at 30-days was significantly higher among patients in S2 vs. S1 (34.6% vs. 16.3%, p = 0.001) (Fig. 3g). This finding was consistent when patients with indeterminate 30-day vital status were considered deceased (39.7% vs. 25.6%, p = 0.017) and in multivariable models adjusted for age, sex, duration of illness and multiple indices of clinical severity (Additional file 1: Table E6). In-hospital outcome and discharge functional status among hospital survivors were also significantly worse among patients in S2 (Fig. 3g). When patients in the both the discovery and internal validation cohorts were pooled and stratified by key pathogen groups, estimates of mortality at 30-days were consistently higher among patients in S2 (Fig. 3h).

### Immune subtypes are differentiated primarily by severe HIV-associated TB

Although we observed a range of infections across both subtypes, the prevalence of severe HIV-related infections, including disseminated HIV-associated TB (indicated by positive urine TB-LAM testing) [28], was significantly and consistently higher in S2 compared to S1 (Fig. 3e, f, Additional file 1: Tables E2-E3). Among patients in the discovery and internal validation cohorts with positive urine TB-LAM testing, those in S2 had higher grades of band intensity (median 3 [IQR; 1–3] vs. 1 [IQR 1–3]), which may correspond to higher mycobacterial loads [29, 30].

### Unsupervised clustering of whole-blood RNA-sequencing data reinforces a two-host subtype partition differentiated by pro-inflammatory innate immune activation, T-cell exhaustion, aberrant NK-cell expansion, and metabolic reprogramming

**Fig. 4:**
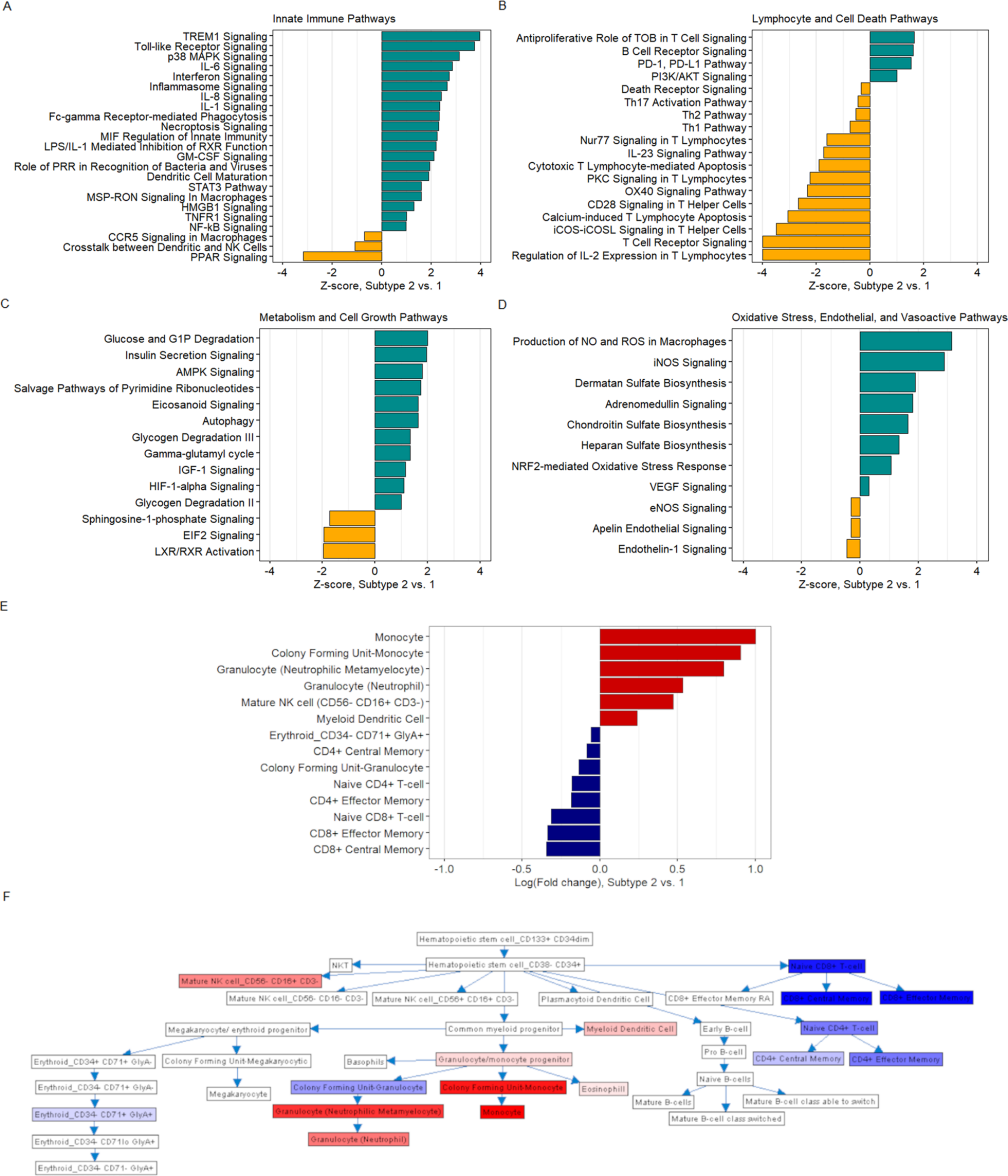
Biological pathway analysis and immune cell-type deconvolution of transcriptional subtypes. **a–d** Ingenuity Pathway Analysis of canonical signaling gene sets differentially enriched across transcriptional subtypes based on log-fold change ≥|1| and Benjamini–Hochberg adjusted p-value ≤ 0.01; Z-score indicates up- versus down-regulation of signaling gene sets in subtype 2 vs. 1 (N = 128). **e** Relative quantities of immune cell-types inferred across subtypes based on ImmQuant digital cell quantification deconvolution; red shading indicates a higher inferred quantity of cell-type in subtype 2 vs. 1 based on log-fold change; blue shading indicates a lower inferred quantity of cell-type in subtype 2 vs. 1 based on log-fold change (N = 128). **f** Hematopoietic lineage plot with relative quantities of immune cell-types inferred across subtypes based on ImmQuant digital cell quantification deconvolution (N = 128); intensity of red shading indicates a higher inferred quantity of cell-type in subtype 2 vs. 1 based on log-fold change; intensity of blue shading indicates a lower inferred quantity of cell-type in subtype 2 vs. 1 based on log-fold change

In unsupervised hierarchical clustering of whole-blood RNA-sequencing data (N = 128), a two-cluster (i.e. transcriptional subtype) model was determined to be optimal (Additional file 1: Figure E7A); 3,561 genes were differentially expressed across subtypes (Additional file 1: Figure E7B). Compared to transcriptional subtype 1 (T1), transcriptional subtype 2 (T2) was characterized by increased expression of genes involved in pathogen recognition, pro-inflammatory cytokine and chemokine signaling, necroptosis, and inflammasome signaling and migration (Fig. 4a). Similar to soluble mediator-derived S2, this marked pro-inflammatory response was accompanied by activation of transcription factor genes (STAT3, PPAR) integral to compensatory, anti-inflammatory immune dampening. Concomitantly, we observed consistent evidence of T-cell exhaustion in T2, including increased expression of genes corresponding to PD-1/PD-L1 inhibitory checkpoint signaling and decreased expression of genes integral in T-cell receptor signaling (Nur77), activation (CD28, iCos-iCosL, PKC), and proliferation (OX40), and cytotoxic T-cell-mediated apoptosis (Fig. 4b).

Quantitatively, immune cell-type deconvolution analysis inferred increased quantities of pro-inflammatory phagocytes and CD56(-)/CD16( +) NK-cells in T2, with concomitant CD4 and CD8 T-cell depletion (Fig. 4e, f). Notably, CD56(-)/CD16( +) NK-cells are an aberrant, likely exhausted subset that expand during HIV-infection and exhibit limited capacity for cytotoxicity and cytokine (i.e. IFN-γ) production, while retaining capability to secrete pro-inflammatory chemokines such as MIP-1β/CCL4 [31, 32].

Metabolically, patients in T2 showed evidence of arrested cell growth and autophagy (Fig. 4c), with concomitant hypoxia-mediated metabolic reprogramming including a switch to glycolysis and upregulation of bioactive, largely pro-inflammatory lipid mediators (eicosanoids, sphingosine-1-phosphate). Patients in T2 also showed increased expression of genes implicated in oxidative stress (NRF2), endothelial dysfunction and angiogenesis (inducible nitric oxide [iNO], VEGF), with decreased expression of genes corresponding to microvascular-protective endothelial NO (Fig. 4d). In T2, which had higher prevalence of shock, we also observed decreased expression of genes involved in maintaining vascular tone (endothelin-1, apelin, adrenomedullin), with increased expression of genes involved in restoration and maintenance of the endothelial glycocalyx (heparan, dermatan, chondroitin sulfates).

### Transcriptional subtypes are differentiated by severe HIV-associated TB, organ dysfunction, and mortality

**Fig. 5:**
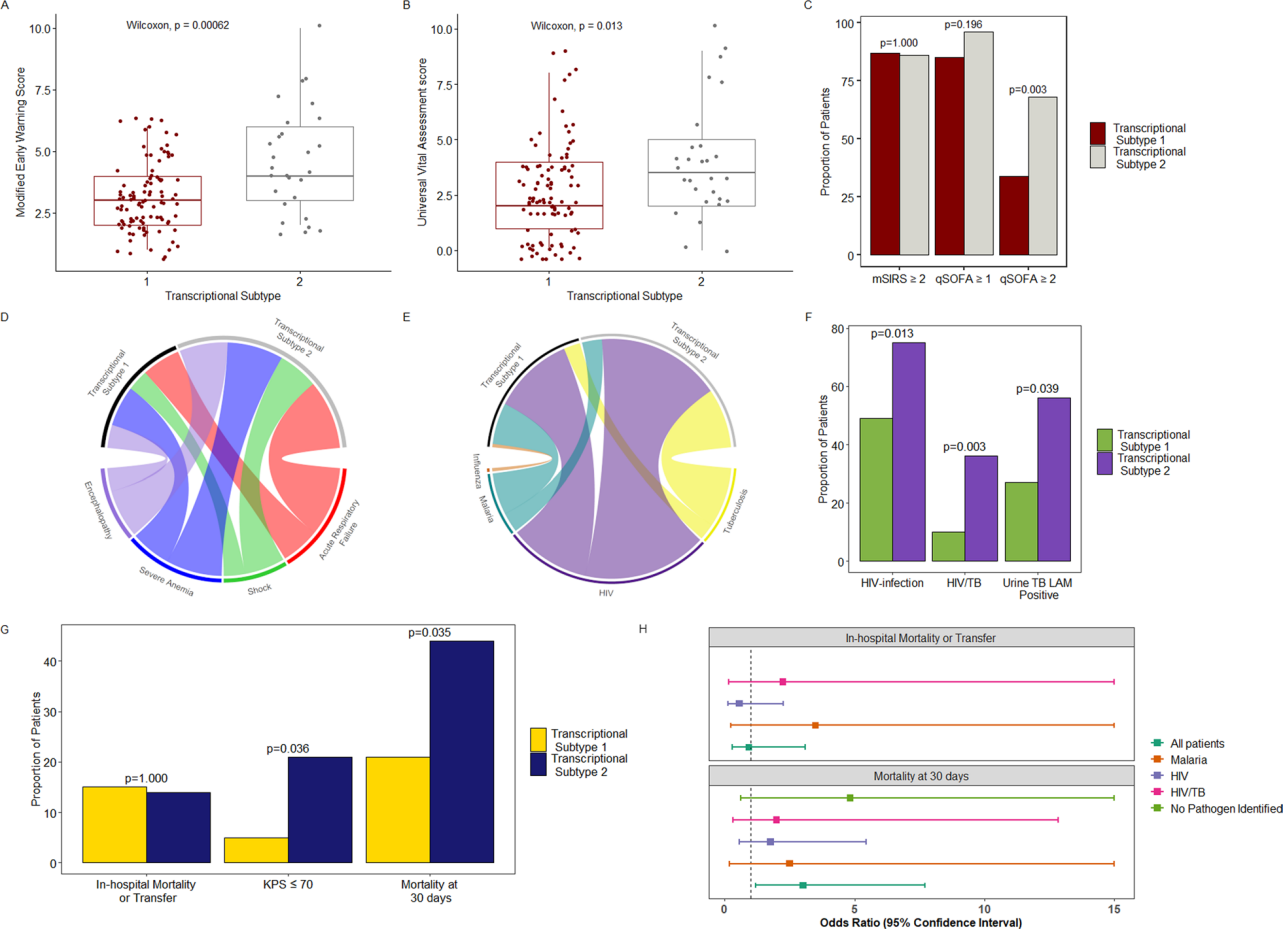
Illness severity scores, distributions of organ failures and pathogens, and outcomes stratified by transcriptional subtypes. **a–c** Modified Early Warning Score, Universal Vital Assessment score, modified systemic inflammatory response syndrome [mSIRS], and quick Sepsis-related Organ Failure assessment [qSOFA] scores stratified by transcriptional subtype; p-values in 5C represent Chi-squared test with continuity correction (N = 128). **d** Chord plot indicating proportion of patients with specific organ failures across each transcriptional subtype; a wider chord band indicates a greater proportion of patients with each corresponding organ failure (N = 128, proportions in subtype 2 vs. 1 as follows: shock: 21.4% vs. 11.0%; acute respiratory failure: 35.7% vs. 20.0%; severe anemia: 28.6% vs. 21.0%; encephalopathy: 25.0% vs. 12.0%). **e** Chord plot indicating proportion of patients with specific infections across each transcriptional subtype; a wider chord band indicates a greater proportion of patients with each corresponding infection (N = 128, proportions in subtype 2 vs. 1 as follows: HIV: 75.0% vs. 48.5%; tuberculosis: 35.7% vs. 10.0%; malaria: 11.5% vs. 24.5%; influenza: 0.0% vs. 2.4%). **f** Proportions of patients with HIV-infection (N = 127), HIV-associated TB (N = 127), and positive urine TB-LAM results (among those tested, N = 55) across each transcriptional subtype. **g** In-hospital outcome (N = 128), impaired functional status [Karnofsky Performance Status; KPS] (N = 108) among hospital survivors, and 30-day vital status across each transcriptional subtype (N = 117); p-values in 5F and 5G represent Chi-squared test with continuity correction. **h** Forest plot indicating univariable (unadjusted) odds ratios for in-hospital outcome and 30-day mortality among patients in transcriptional subtype 2 vs. subtype 1, stratified by key pathogen groups [patients with influenza omitted given small number of events in that pathogen group, odds ratio for in-hospital outcome omitted for patients with no pathogen detected as all events in transcriptional subtype 1; for visualization, upper limit of 95% confidence interval for in-hospital outcome truncated at 15 for patients with HIV-associated TB (upper limit 29.76) and malaria (upper limit 51.46), as well as for 30-day outcome for patients with no pathogen identified (upper limit 38.39) and malaria (upper limit 34.67)

Consistent with our immune mediator analyses, physiologic derangement was more severe in T2, as indicated by higher MEWS and UVA scores and a greater proportion of patients with qSOFA score ≥ 2 (Fig. 5a–c, Additional file 1: Table E7). Compared to T1, patients in T2 had higher prevalence of shock, acute respiratory failure, severe anemia, and encephalopathy (Fig. 5d, Additional file 1: Table E7). Patients assigned to T2 were predominantly HIV-infected with advanced immunosuppression (HIV clinical stage 3 or 4), severe and often disseminated TB, and less frequent malaria (Fig. 5e, f, Additional file 1: Table E7). Similar to immune mediator subtypes, patients in T2 with positive TB-LAM results had higher band grade intensities than those in T1 (median 3 [IQR 1–3] vs. 1 [IQR 1–1]).

Mortality at 30-days was significantly higher among patients assigned to T2 vs. T1 (44.0% vs. 20.7%, p = 0.035) (Fig. 5g, Additional file 1: Table E8). This finding was consistent when patients with indeterminate 30-day vital status were considered deceased (50.0% vs. 27.0%, p = 0.038), and in multivariable models adjusted for age, sex, illness duration and severity (Additional file 1: Table E8). For patients discharged alive, those in T2 were significantly more likely to have impaired functional status (Fig. 5g). When patients were stratified by pathogen groups, estimates of mortality at 30-days were consistently higher among those in T2 (Fig. 5h).

### Subtype-specific prevalence of malaria varies by immune compartment but is consistent for HIV-associated TB

Among the 122 patients for whom both soluble mediator and RNA-sequencing data were available, 75 (62%) were assigned to mediator- and transcriptionally-derived subtypes with similar immunological features (i.e. consistently assigned to relatively hyperinflammatory [S2, T2] vs. hypo-inflammatory [S1, T1] immune mediator and transcriptional subtypes) and 47 (38%) were assigned to dissimilar subtypes (Additional file 1: Figure E8A, Table E9). Although the proportion of HIV-infected patients with more advanced immunosuppression (clinical stages 3 and 4) was highest in T2, prevalence of severe HIV-related infections, including HIV-associated TB, was consistently higher in mediator-derived and transcriptional hyperinflammatory subtypes (Additional file 1: Tables E2-E3, E9 and Figures E8B). In contrast, the prevalence of malaria varied substantially across each approach, with malaria prevalence of 24.5–40% in mediator-derived hyperinflammatory subtypes (S2) and 11.5% in the hyperinflammatory, T-cell exhausted transcriptional subtype (T2) (Additional file 1: Table E7 and Figure E8C).

## Discussion

Among adults hospitalized with suspected sepsis in Uganda, we identified distinct immune subtypes defined by differential activation of pro-inflammatory innate and adaptive immune pathways, with transcriptional evidence of T-cell exhaustion, aberrant NK-cell expansion, and hypoxia-induced metabolic reprogramming accompanying the hyperinflammatory subtype. Immune subtypes defined by upregulation of these pathways were consistently associated with severe and often disseminated HIV-associated TB as well as more extensive organ dysfunction and worse clinical outcomes. Our results highlight unique host- and pathogen-driven features of sepsis immunopathology in sub-Saharan Africa, and reinforce the need to develop and test more biologically-informed treatment strategies in the region, including those incorporating immunomodulation.

Despite advances towards identifying more precise therapeutic targets and higher-risk, treatment-responsive sepsis subgroups in HICs, biological heterogeneity inherent to sepsis in low-income-settings remains unexplored [5–10, 33, 34]. Here, we show that sepsis host response subtypes differentiated by activation of key immunometabolic pathways are present in a generalizable SSA setting and associated with unique microbiological and prognostic features. If replicated elsewhere, these host subtypes could conceivably be leveraged to inform more locally-relevant models of sepsis immunopathology and targeted immunomodulatory therapies. For example, while over 50 clinical trials of corticosteroids in sepsis and septic shock have been conducted in HICs (primarily in the context of mitigating potential adrenal insufficiency), none have been conducted in SSA, with guidance on their use in low-income settings driven by expert opinion [35, 36]. Accordingly, there is a need to further evaluate the role of these low-cost immunomodulatory agents in sepsis management in SSA, in parallel with continued efforts to precisely define therapeutically exploitable pathways.

In HICs, studies comparing sepsis host response profiles among patients with different causative pathogens have reported conflicting results [37]. While several have found host profiles to be consistent across varied microbiological etiologies and sites of infection [37–39], others identified source-specific heterogeneity in the host response [40]. Across soluble mediator- and transcriptionally-derived immune subtypes identified in our Ugandan cohort, the prevalence of severe HIV-related infections, including frequently disseminated TB, was consistently higher in those defined by greater pro-inflammatory innate immune activation, aberrant NK-cell expansion, and T-cell exhaustion. Despite the disproportionate burden of sepsis among HIV-infected adults in SSA, little remains known about the immunopathology of HIV-associated sepsis in the region, of which disseminated TB is a leading cause [3, 41, 42]. Independent of critical illness, HIV-infected adults in SSA show evidence of persistent monocyte/macrophage activation and systemic inflammation, some of which persists despite antiretroviral therapy (ART) and viral suppression [43]. HIV-related activation of monocytes/macrophages may also induce of a state of hyperresponsive immune priming that precipitates disproportionate release of pro-versus anti-inflammatory cytokines following pathogen stimulation [32]. Collectively, our observations of a high-risk, HIV-predominant host subtype defined by amplified pro-inflammatory innate immune activation and NK- and T-cell exhaustion highlight the need to evaluate the role of immunomodulatory agents in conjunction with prompt initiation or continuation of ART among patients with HIV-associated sepsis in SSA, in parallel with efforts to optimize antimicrobial strategies for TB and other high-burden co-infections [44, 45].

Although the prevalence of severe HIV-associated infections was consistently higher in immune mediator and transcriptionally-derived hyperinflammatory subtypes, subtype-specific prevalence of malaria varied, with higher prevalence in mediator-derived hyperinflammatory subtypes. Nonetheless, patients with malaria assigned to hyperinflammatory subtypes, which showed concomitant endothelial dysfunction, had consistently poorer outcomes. While end-organ sequestration due to microvascular obstruction and endothelial dysfunction are established pathobiological mechanisms in severe malaria, the role of inflammatory mediators is less well-defined [46]. Consistent with our data, prior studies have identified imbalances between pro- and anti-inflammatory mediators among patients with severe falciparum malaria at higher risk of organ dysfunction and death [46, 47]. Although prior trials of immunomodulatory agents in severe falciparum malaria have not shown benefit [48], eventual stratification of trial populations based on immune-inflammatory subtypes could conceivably be used to enhance prognostic and predictive enrichment when evaluating new agents [48].

Our study has limitations. First, our findings are derived from a single-center cohort of medical ward patients and require replication in other geographic and clinical care settings in SSA. Second, although serum mediator samples were available for nearly all patients (96%), whole-blood RNA samples were available from approximately 43%. Third, as we were unable to isolate peripheral-blood-mononuclear-cells given resource-limitations at our study site, we performed computational deconvolution analyses to infer quantities of immune-cell subsets. Next, although rapid diagnostics for key pathogens were performed in the parent study based on WHO guidelines, we did not perform blood cultures. Lastly, the cross-sectional nature of our sample collection precludes determination of temporal stability of the identified host subtypes.

## Conclusions

We have demonstrated the presence of immunopathologically-distinct and clinically-meaningful host response subtypes among adults with suspected sepsis in Uganda. In conjunction with improvements in acute-care capacity, future studies are needed to refine understanding of sepsis immunopathology in SSA with the goal of developing more locally-relevant, biologically-informed, and clinically-effective management strategies.

## Supplementary Information

**Additional file 1:** Supplemental methods, tables, and figures.

## Data Availability

RNA-sequencing data analyzed in this study are available in the NIH National Center for Biotechnology Information Sequence Read Archive under BioProject accession number PRJNA794277. Other de-identified data will be made available to researchers affiliated with an appropriate institution following mutual signing of a data access agreement and obtainment of necessary ethics approvals. All code is available on request from Drs. Cummings and Price.

## Abbreviations

SSA: Sub-Saharan Africa
HIC: High-income countries
EGRH: Entebbe General Referral Hospital
RESERVE-U: Research in the Epidemiology of Severe and Emerging Infections in Uganda
WHO: World Health Organization
IL: Interleukin
IFN: Interferon
IP-10/CXCL10: IFN-γ-induced-protein-10/C-X-C motif-chemokine-10
MIP-1α/CCL3: Macrophage-inflammatory-protein-1-alpha/chemokine (C–C motif)-ligand 3
MIP-1β/CCL4: Macrophage-inflammatory-protein-1-beta/chemokine (C–C motif) ligand-4
TNF-α: Tumor-necrosis-factor-α
Ang: Angiopoietin
MIF: Macrophage-migration-inhibitory-factor
sTNFR1: Soluble-TNF-receptor-type-1
sIL-2Ra/sCD25: Soluble-IL-2-receptor-alpha/soluble-CD25
PAI-1: Plasminogen-activator-inhibitor-1
RNA: Ribonucleic acid
qSOFA: Quick-Sepsis-related Organ Failure Assessment
S1: Soluble mediator subtype 1
S2: Soluble mediator subtype 2
UVA: Universal Vital Assessment
MEWS: Modified Early Warning Score
TB: Tuberculosis
TB-LAM: Tuberculosis-Lipoarabinomannan
T1: Transcriptional subtype 1
T2: Transcriptional subtype 2
ART: Anti-retroviral therapy
