# Supplementary material for: Multidimensional analysis of the host response reveals prognostic and pathogen-driven immune subtypes among adults with sepsis in Uganda
Source: Crit Care. 2022 Feb 8;26:36. doi: 10.1186/s13054-022-03907-3 (PMC8822787; doi:10.1186/s13054-022-03907-3)
Supplement: Supplementary file 1 — Additional file 1: Supplemental methods, tables, and figures. [file 13054_2022_3907_MOESM1_ESM.pdf]

Supplementary Data

Supplemental Methods

Study Site and Capacity

Entebbe General Referral Hospital (EGRH) is a 200-bed public district referral hospital with a catchment area of approximately 3 million persons. In the primary catchment area, HIV prevalence is approximately 6% and malaria is endemic [11]. Representative of a general district hospital in SSA, there is no intensive care unit at EGRH. No vasopressor or inotropic agents are available on the hospital wards and intravenous (IV) fluid is typically delivered as 250-500 milliliter infusions of crystalloid, either normal saline or Ringer's lactate solution. As no piped oxygen was available at EGRH during the study period, oxygen concentrators were provided to hospital wards as part of the study program.

RESERVE-U Enrollment Criteria

Patients were included in the parent RESERVE-U study [11] if they fulfilled the following criteria: (1) age ≥18 years, (2) reported a history of fever or had a recorded axillary temperature of ≥37.5ºC at presentation, (3) had clinical illness severe enough to warrant admission to hospital, and (4) were able to provide informed consent or had a surrogate available to do so. Patients were excluded if they presented following trauma or were admitted to a non-medical ward. During the study period, all admissions to the medical wards were screened for eligibility by study staff. Patients were screened on weekdays during daytime hours and were enrolled as close to admission as possible and no longer than 24 hours afterwards. Although patients 5-17 years of age were also included in the parent RESERVE-U cohort, given variations in pathogen-specific infection risk, normal vital sign parameters, and infection-related host immune responses across age groups, for this analysis we included only adults (age ≥18 years).

Outcomes

The primary outcome of the RESERVE-U study was vital status at 30-days after hospital discharge (obtained via telephone from patients or their surrogates). Secondary outcomes included a composite measure of in-hospital outcome (death in-hospital or transfer to Uganda's national referral hospital due to progressive severity of illness) and functional status at discharge (among patients who survived and were not transferred). Patients transferred to the national hospital were contacted to assess vital status at 30-days.

Clinical Data, Sample Collection, and Rapid Pathogen Diagnostics

Study procedures for the parent RESERVE-U cohort have been described [11]. Briefly, enrollment occurred within 24 hours of hospital admission, at which time all patients underwent clinical assessments and had rapid testing performed for malaria, HIV, and influenza; for HIV-infected patients testing for tuberculosis (TB) was also performed. Testing for these pathogens was informed by World Health Organization Integrated Management of Adolescent and Adult Illness guidelines for sepsis and septic shock in resource-limited hospitals in SSA [12]. These guidelines emphasize rapid testing for malaria and HIV, a low threshold for TB testing among HIV-infected patients, and consideration of testing or empiric treatment for influenza. For malaria, rapid testing was performed at EGRH using qualitative detection of histidine-rich protein II and lactate dehydrogenase of *P. falciparum* in whole-blood using the SD Bioline Malaria AG P.f. platform (Alere/Abbott, Abbott Park, IL, USA). For all patients not known to be HIV infected, HIV testing was performed at EGRH using serial diagnostic platforms (Determine HIV-1/2 Ag/Ab, Alere/Abbott); Chembio HIV 1/2 Stat-Pak, Chembio Diagnostic Systems, Medford, NY, USA), Uni-gold Recombigen HIV-1/2, Trinity Biotech, Ireland). For all enrolled HIV-infected patients (known or newly diagnosed), a single urine sample (obtained via urinary catheter or spontaneous void) and a single spontaneously expectorated sputum sample, if

obtainable, were tested for evidence of *Mycobacterium tuberculosis* (MTB) infection at EGRH by the study laboratory technician. For urine samples, 60µL of unconcentrated urine was tested using the Determine™ TB-LAM Ag assay (Alere/Abbott) as per the manufacturer's recommended operating procedure. The intensity of any visible band on the test strip was graded by comparing it with band intensities on the manufacturer's post-2014 reference card scale; results were considered positive using the grade 1 cutoff. For sputum samples, testing was performed using the Xpert MTB/RIF Ultra platform (Cepheid, Sunnyvale, CA, USA). Sputum smear microscopy was performed at the discretion of treating clinicians as was Xpert MTB/RIF Ultra testing of sputum for non-HIV-infected patients. Nasopharyngeal swab samples were tested for influenza A and B viruses via real-time polymerase-chain-reaction (RT-PCR). Influenza PCR, implemented with primers from the U.S. Centers for Disease Control and Prevention, was performed as soon as possible following sample collection at Uganda Virus Research Institute, located approximately 2 kilometers from EGRH.

At enrollment, peripheral blood samples were collected into vacutainer tubes and centrifuged, with resulting serum samples stored at -80ºC. For a subset of consecutively enrolled patients, whole-blood samples were collected in PAXgene blood RNA tubes (PreAnalytiX, Qiagen/BD, Hombrechtikon, Switzerland) and stored at -80ºC.

<u>Serum Immunoassays</u>

Cryopreserved serum samples collected at the time of enrollment were sent to Eve Technologies (Calgary, Alberta, Canada) on dry ice for analysis. Interleukin (IL)-6, IL-8, IL-10, interferon (IFN)-γ, IFN-γ-induced protein-10/C-X-C motif chemokine 10 (IP-10/CXCL10), macrophage inflammatory protein-1-alpha/chemokine (C-C motif) ligand 3 *(*MIP-1α/CCL3), macrophage inflammatory protein-1-beta/chemokine (C-C motif) ligand 4 (MIP-1β/CCL4), and

tumor necrosis factor-α (TNF-α), angiopoietin-2 (Ang-2), macrophage migration inhibitory factor (MIF), plasminogen activator inhibitor-1 (PAI-1), soluble TNF-receptor type 1 (sTNFR1) and soluble IL-2 receptor alpha/soluble CD25 (sIL-2RA/sCD25) were quantified using custom Luminex 200 system kits (Luminex, Austin, TX, USA) from MilliporeSigma (Burlington, Massachusetts, USA). Angiopoietin-1 (Ang-1) was quantified using a custom Luminex 200 system kit from R&D Systems (Minneapolis, MN, USA). Values below the lower limit of assay quantification (1.1% of all samples) were replaced with the lowest value that could be reliably quantified for that particular mediator. Values above the upper limit of quantification (1.5% of all samples) were replaced with the highest standard curve value for each particular mediator.

Whole-blood RNA isolation, library preparation, and sequencing

From cryopreserved whole-blood samples collected in PAXgene blood RNA tubes (PreAnalytiX, Qiagen/BD, Hombrechtikon, Switzerland) at the time of study enrollment, RNA was isolated and purified using PAXgene blood RNA kits (Qiagen, Hilden, Germany). RNA sample quantity and integrity were assessed using Qubit 2.0 Fluorometer (Invitrogen, Thermo Fisher Scientific, Waltham, MA, USA) and 4200 TapeStation (Agilent Technologies, Palo Alto, CA, USA) platforms, respectively. DNase treatment was performed using Turbo DNase (Thermo Fisher Scientific) prior to library preparation. rRNA depletion was performed using QIAseq FastSelect – rRNA/Globin Kit (Qiagen). RNA sequencing library preparation was performed using the NEBNext Ultra RNA Library Prep Kit for Illumina following the manufacturer's recommendations (NEB, Ipswich, MA, USA). Sequencing libraries were validated using the 4200 Tapestation (Agilent Technologies) and quantified using the Qubit 2.0 Fluorometer (Invitrogen, Thermo Fisher Scientific) as well as by quantitative PCR (Applied Biosystems, Thermo Fisher Scientific). Sequencing libraries were multiplexed and clustered on one lane of a flowcell and loaded on the Illumina HiSeq 4000 instrument (Illumina, Inc., San Diego, CA, USA) according to

manufacturer's instructions. Samples were sequenced using a 2x150 paired end configuration. Image analysis and base calling were conducted by the HiSeq control software. Raw sequence data were converted into FASTQ files and de-multiplexed using Illumina's bcl2fastq 2.17 software. One mis-match was allowed for index sequence identification. Adapters were trimmed from raw reads using Trimmomatic [13] and RNA-sequencing data quality were assessed with FastQC [14]. Sequencing reads were aligned to the human genome (GRCh38) using STAR [15] and transcript quantification was performed using the R-subread package's featureCounts utility [16].

Analysis of Soluble Immune Mediators

To identify and characterize immune subtypes using soluble host mediators, we performed unsupervised clustering followed by a series of unsupervised and supervised classification, regression, and network analyses. First, mediator concentrations were $\log_{10}$ transformed to minimize skew, with resulting distributions assessed visually using histogram and Q-Q plots. In preparation for clustering and principal component analyses, mediator data were then centered and scaled to a mean of zero and standard deviation of one. Using Ward's method, Euclidean distance, and a range of clusters (*k*) set from *k*=2 to *k*=12, we first applied agglomerative hierarchical clustering to $\log_{10}$ transformed, scaled, and centered serum mediator concentrations to initiate a cluster partition, followed by a k-means procedure to consolidate cluster membership (*FactoMineR* R package) [18]. We used within-cluster sum-of-squares to determine the optimal number of clusters, which we confirmed using over 20 indices of cluster partition validity generated by the *NbClust* R package [19]. We visualized patient-level, between-cluster variance in mediator concentrations using principal component analysis (PCA) (*FactoMineR* and *factoextra* R packages) and standardized heatmaps (*ComplexHeatmap* R package). Given our unsupervised approach and to avoid overfitting, we applied our clustering

strategy to the randomly partitioned discovery and internal validation cohorts (generated using the *caTools* R package) independently.

After determining the optimal number of clusters (i.e. immune subtypes), we used several methods to explore subtype-specific molecular signatures in the discovery cohort. First, to infer the most influential variables driving subtype separation, we determined representation of mediator variables on the first two principal components by calculating and plotting squared factor loadings for each variable (*FactoMinerR, factoextra,* and *corrplot* R packages). Separately, we applied a gradient-boosted decision tree classifier model (*XGBoost* R package), trained to predict subtype assignment, to our mediator variables, and identified the most important discriminatory variables using their respective split gain values. Optimal hyperparameters (learning rate [eta], tree depth [max_depth], and number of trees [nround]) for the model were determined using 10-fold cross validation in the *caret* R package, with the remaining hyperparameters left at default settings. Next, to explore longitudinal, subtype-specific changes in soluble mediators over the course of illness, we evaluated the relationship between mediator concentrations and reported duration of illness at admission (obtained via patient or surrogate) using robust regression (*ggplot2* and *MASS* R packages), with patient-level datapoints stratified by cluster assignment. Lastly, to determine inter-mediator relationships within each discovery cohort cluster and identify "central" mediators around which each cluster may be coordinated, we constructed force-directed weighted correlation networks based on the Fruchterman-Reingold method (*qgraph* R package) [20-22]. Each mediator variable was set as a network node with between-mediator correlations significant at p-value ≤0.05 indicated by weighted edges. To identify the core, most "central" mediators within each cluster, metrics of network centrality were calculated for each mediator node [20,21]. These included strength (also known as degree centrality, with higher values indicating that a node is involved in large

number of strong interactions, in this case, correlation weights), closeness (a measure of spatial distance to other nodes, with higher values indicating greater spatial proximity, implying that such nodes can interact more rapidly with others), and betweenness (a measure of a node's role in connecting other nodes in a network, with greater values indicating that a node frequently acts as an "intermediary path" between others). Nodes with ≥1 metric above the standardized cluster mean (z-score >0) were considered central [20,21].

Analysis of whole-blood RNA-sequencing data

Independent of immune mediator analyses, we performed unsupervised clustering of whole-blood RNA sequencing data from a subset of consecutive patients in the RESERVE-U cohort. Following normalization and removal of transcripts with very low expression (<100 reads), transcripts were first ranked by median absolute variance across all samples, with the top 5,000 selected for hierarchical clustering, which we performed using the same methods as those applied to immune mediators (*FactoMinerR* R package) [18]. Following determination of the optimal number of transcriptional clusters using within-cluster sum-of-squares, differential gene expression analysis was performed using the *DESeq2* and *DEvis* R packages to identify genes that were differentially expressed across each cluster [23,24]. Genes were determined to be differentially expressed based on a log-fold change ≥|1| and Benjamini-Hochberg adjusted p-value ≤0.01. Differentially expressed gene sets were selected for biological pathway analyses (Ingenuity Pathway Analysis, Qiagen, Hilden, Germany), results of which were examined to infer functional differences between identified clusters. Using the ImmQuant software package, we applied cell-type deconvolution to our differential expressed gene sets to infer relative immune cell quantities across the identified transcriptional clusters [25]. ImmQuant employs an established global digital cell quantification method which infers changes in the relative quantity of specific immune cell subsets across groups based on the IRIS and DMAP compendiums

[26,27]. Derived from purified cell populations, these curated datasets contain mRNA subtypes associated with distinct hematopoietic lineages across a range of cell maturation and differentiation states.

Statistical Analyses

In presentations of clinical data, continuous variables are expressed as medians (interquartile range [IQR]) and categorical variables are summarized as counts and percentages with 95% confidence intervals (CI) and two-sided p-values presented where relevant. Clinical and microbiological characteristics across immune mediator- and transcriptional subtypes were compared using Chi-squared, Fisher exact, or Wilcoxon rank sum tests, as appropriate. Univariable and multivariable logistic regression was also used to compare the primary outcome of 30-day mortality. Given the exploratory nature of this study, no adjustment for multiple comparisons was performed unless indicated. For the primary outcome of 30-day vital status, mortality was not imputed if vital status was unknown, and a sensitivity analysis was performed to account for potential bias due to loss-to-follow-up.

Supplemental Tables

Table E1: Characteristics of patients with and without RNA-sequencing data

Patient characteristic	All patients (N=301)	RNA-sequencing (N=128)	No RNA-sequencing (N=173)
Female sex, n (%)	178/301 (59.1)	80/128 (62.5)	98/173 (56.6)
Age, years, median [IQR]	32 [26,42]	33 [26,43]	32 [26,41]
Duration of illness prior to admission, days, median [IQR][a]	4 [3,7]	4 [3,7]	4 [3,7]
History of fever, n (%)	301/301 (100)	128/128 (100)	173/173 (100)
Night sweats, n (%)	236/301 (78.4)	99/128 (77.3)	137/173 (79.2)
Headache, n (%)	238/301 (79.1)	104/128 (81.3)	134/173 (77.5)
Cough, n (%)	188/301 (62.5)	72/128 (56.3)	116/173 (67.1)
Diarrhea, n (%)	102/301 (33.9)	48/128 (37.5)	54/173 (31.2)
Shortness of breath, n (%)	68/301 (22.6)	29/128 (22.7)	39/173 (22.5)
Dysuria, n (%)	40/301 (13.3)	15/128 (11.7)	25/173 (14.5)
Received antibiotic or antimalarial agent prior to admission, n (%)	107/301 (35.5)	45/128 (35.2)	62/173 (35.8)
Temperature ≥38°C, n (%)	107/301 (35.5)	47/128 (36.7)	60/173 (34.7)
Temperature <36°C, n (%)	87/301 (28.9)	44/128 (34.4)	43/173 (24.9)
Heart rate, beats/min, median [IQR]	98 [87,109]	97 [85,110]	99 [88,108]
Respiratory rate, beats/min, median [IQR]	22 [21,26]	22 [22,27]	22 [20,24]
Systolic blood pressure, mmHg, median [IQR]	103 [91,117]	106 [95,118]	101 [91,115]
Oxygen saturation, %, median [IQR]	97 [95,98]	97 [96,98]	97 [95,98]
Encephalopathy, n (%)[b]	58/301 (19.3)	19/128 (14.8)	39/173 (22.5)
qSOFA score ≥2, n (%)[c]	134/301 (44.5)	53/128 (41.4)	81/173 (46.8)
qSOFA score ≥1, n (%)[c]	262/301 (87.0)	112/128 (87.5)	150/173 (86.7)
Modified SIRS score ≥2, n (%)[d]	257/301 (85.4)	111/128 (86.7)	146/173 (84.4)
MEWS, median [IQR]	3 [2,5]	3 [2,4]	3 [2,5]
UVA score, median [IQR]	2 [1,4]	3 [2,4]	2 [1,4]
Shock, n (%)[e]	41/301 (13.6)	17/128 (13.3)	24/173 (13.9)
Acute respiratory failure, n (%)[f]	65/301 (21.6)	30/128 (23.4)	35/173 (20.2)
Severe anemia, n (%)[g]	60/301 (19.9)	29/128 (22.7)	31/173 (17.9)
HIV-infected, n (%)	157/299 (52.5)	69/127 (54.3)	88/172 (51.2)
WHO clinical stage 3 or 4, n (%)	126/157 (80.3)	51/69 (73.9)	75/88 (85.2)
Newly diagnosed HIV infection, n (%)	21/157 (13.4)	13/69 (18.8)	8/88 (9.1)
On ART prior to admission, n (%)[h]	92/136 (67.6)	38/56 (67.9)	54/80 (67.5)
On TMP-SMX prior to admission, n (%)[h]	96/136 (70.6)	43/56 (76.8)	53/80 (66.3)
Malaria RDT positive, n (%)	61/296 (20.6)	27/124 (21.8)	34/172 (19.8)
Microbiological TB positive, n (%)[i]<br>Urine TB-LAM positive	52/301 (17.3)<br>41/124 (33.1)	20/128 (15.6)<br>20/55 (36.4)	32/173 (18.5)<br>21/69 (30.4)
Influenza PCR positive, n (%)	17/272 (6.3)	2/108 (1.9)	15/164 (9.1)
Death in-hospital or transfer, n (%)	42/301 (14.0)	19/128 (14.8)	23/173 (13.3)
Duration of hospitalization, days, median [IQR][j]	5 [3,7]	5 [4,7]	5 [3,8]
KPS ≤70 at alive discharge, n (%)	20/257 (7.8)	9/108 (8.3)	11/149 (7.4)
Death at 30-days post-discharge, n (%)	63/272 (23.2)	30/117 (25.6)	33/155 (21.3)

Abbrevations: IQR: interquartile range, qSOFA: quick Sequential (Sepsis-related) Organ Failure Assessment, SIRS: Systemic Inflammatory Response Syndrome, MEWS: Modified Early Warning Score, UVA: universal vital assessment, HIV: human immunodeficiency virus, WHO: World Health Organization,

ART: anti-retroviral therapy, RDT: rapid diagnostic test, TB: tuberculosis, LAM: lipoarabinomannan, PCR: polymerase chain reaction; KPS: Karnofsky performance status.

Legend: [a]Unknown for 1 patient, [b]Anything other than “Alert” on AVPU (alert, responsive to voice, responsive to pain, unresponsive) mental status assessment; [c]Systolic blood pressure ≤100mmHg, respiratory rate ≥22 breaths/min, and encephalopathy, latter defined using AVPU scale; [d]Temperature ≥38°C or <36°C, heart rate ≥90 beats/min, respiratory rate ≥20 breaths/min; [e]Systolic blood pressure ≤90 mmHg despite administration of ≥1 liter of intravenous fluid; [f]Oxygen saturation ≤90% or respiratory rate ≥30 breaths/min; [g]Hemoglobin <9 g/dl or administration of blood transfusion. [h]Denominator is number with known HIV-infection prior to admission; [i]Sputum Xpert Ultra or smear positive or urine TB-LAM positive; [j]Unknown for 11 patients.

Table E2: Patient characteristics in the discovery cohort stratified by immune subtype

Patient characteristic	All patients (N=201)	Subtype 1 (N=105)	Subtype 2 (N=96)	p-value[a]
Female sex, n (%)	116/201 (57.7)	63/105 (60.0)	53/96 (55.2)	0.586
Age, years, median [IQR]	32 [26,40]	32 [26,40]	32 [27,41]	0.655
Duration of illness prior to admission, days, median [IQR][b]	4 [3, 7]	4 [2, 7]	5 [3, 7]	0.123
History of fever, n (%)	201/201 (100.0)	105/105 (100.0)	96/96 (100.0)	--
Night sweats, n (%)	161/201 (80.1)	78/105 (74.3)	83/96 (86.5)	0.047
Headache, n (%)	158/201 (78.6)	79/105 (75.2)	79/96 (82.3)	0.296
Cough, n (%)	125/201 (62.2)	62/105 (59.0)	63/96 (65.6)	0.415
Diarrhea, n (%)	70/201 (34.8)	26/105 (24.8)	44/96 (45.8)	0.003
Shortness of breath, n (%)	47/201 (23.4)	20/105 (19.0)	27/96 (28.1)	0.176
Dysuria, n (%)	31/201 (15.4)	10/105 (9.5)	21/96 (21.9)	0.026
Received antibiotic or antimalarial agent prior to admission, n (%)	70/201 (34.8)	34/105 (32.4)	36/96 (37.5)	0.540
Temperature ≥38°C, n (%)	76/201 (37.8)	28/201 (26.7)	48/96 (50.0)	0.001
Temperature <36°C, n (%)	58/201 (28.9)	36/105 (34.3)	22/96 (22.9)	0.105
Heart rate, beats/min, median [IQR]	98 [86, 108]	95 [83, 105]	100 [89, 114]	0.015
Respiratory rate, beats/min, median [IQR]	22 [21, 26]	22 [20, 24]	22 [22, 26]	0.095
Systolic blood pressure, mmHg, median [IQR]	104 [91, 118]	105 [98, 122]	99 [90, 112]	0.010
Oxygen saturation, %, median [IQR]	97 [96, 98]	97 [96, 98]	97 [95, 98]	0.812
Encephalopathy, n (%)[c]	40/201 (19.9)	16/105 (15.2)	24/96 (25.0)	0.120
qSOFA score ≥2, n (%)[d]	88/201 (43.8)	37/105 (35.2)	51/96 (53.1)	0.016
qSOFA score ≥1, n (%)[d]	174/201 (86.6)	88/105 (83.8)	86/96 (89.6)	0.321
Modified SIRS score ≥2, n (%)[e]	173/201 (86.1)	88/105 (83.8)	85/96 (88.5)	0.445
MEWS, median [IQR]	3 [2, 4]	3 [2, 4]	4 [2, 5]	<0.001
UVA score, median [IQR]	3 [1, 4]	2 [0, 4]	3 [2, 4]	0.012
Shock, n (%)[f]	28/201 (13.9)	11/105 (10.5)	17/96 (17.7)	0.202
Acute respiratory failure, n (%)[g]	39/201 (19.4)	18/105 (17.1)	21/96 (21.9)	0.504
Severe anemia, n (%)[h]	39/201 (19.4)	14/105 (13.3)	25/96 (26.0)	0.036
HIV-infected, n (%)	106/199 (53.3)	43/103 (42.0)	63/96 (65.6)	0.001
WHO clinical stage 3 or 4, n (%)	91/106 (85.8)	37/43 (86.0)	54/63 (85.7)	0.962
Newly diagnosed HIV-infection, n (%)	12/106 (11.3)	3/43 (7.0)	9/63 (14.3)	0.353
On ART prior to admission, n (%)[i]	63/94 (67.0)	29/40 (72.5)	34/54 (63.0)	0.331
On TMP-SMX prior to admission, n (%)[i]	65/94 (69.1)	30/40 (75.0)	35/54 (64.8)	0.290
Malaria RDT positive, n (%)	38/197 (19.2)	15/103 (14.6)	23/94 (24.5)	0.078
Microbiological TB positive, n (%)[j]<br>Urine TB-LAM positive	35/201 (17.4)<br>27/83 (32.5)	9/105 (8.6)<br>5/31 (16.1)	26/96 (27.1)<br>22/52 (42.3)	0.001<br>0.016
Influenza PCR positive, n (%)	14/184 (7.6)	10/95 (10.5)	4/89 (4.5)	0.123
Death in-hospital or transfer, n (%)	28/201 (13.9)	10/105 (9.5)	18/96 (18.8)	0.092
Duration of hospitalization, days, median [IQR][k]	5 [3, 7]	5 [3, 6]	5 [3, 8]	0.692
KPS ≤70 at alive discharge, n (%)	12/173 (6.9)	4/95 (4.2)	8/78 (10.3)	0.141
Death at 30-days post-discharge, n (%)	44/179 (24.6)	15/91 (16.5)	29/88 (33.0)	0.011

Abbrevations: IQR: interquartile range, qSOFA: quick Sequential (Sepsis-related) Organ Failure Assessment, SIRS: Systemic Inflammatory Response Syndrome, MEWS: Modified Early Warning Score, UVA: universal vital assessment, HIV: human immunodeficiency virus, WHO: World Health Organization, ART: anti-retroviral therapy, RDT: rapid diagnostic test, TB: tuberculosis, LAM: lipoarabinomannan, PCR: polymerase chain reaction; KPS: Karnofsky performance status.

Legend: [a]Fisher Exact or Chi-squared test with continuity correction; [b]Unknown for 1 patient; [c]Anything other than “Alert” on AVPU (alert, responsive to voice, responsive to pain, unresponsive) mental status assessment; [d]Systolic blood pressure ≤100mmHg, respiratory rate ≥22 breaths/min, and encephalopathy,

latter defined using AVPU scale; [e]Temperature ≥38°C or <36°C, heart rate ≥90 beats/min, respiratory rate ≥20 breaths/min; [f]Systolic blood pressure ≤90 mmHg despite administration of ≥1 liter of intravenous fluid; [g]Oxygen saturation ≤90% or respiratory rate ≥30 breaths/min; [h]Hemoglobin <9 g/dl or administration of blood transfusion. [i]Denominator is number with known HIV-infection prior to admission; [j]Sputum Xpert Ultra or smear or urine TB-LAM positive; [k]Unknown for 7 patients.

Table E3: Patient characteristics in the validation cohort stratified by immune subtype

Patient characteristic	All patients (N=87)	Subtype 1 (N=67)	Subtype 2 (N=20)
Female sex, n (%)	55/87 (63.2)	44/67 (65.7)	11/20 (55.0)
Age, years, median [IQR]	32 [27, 43]	31 [26, 43]	36 [28, 42]
Duration of illness prior to admission, days, median [IQR]	4 [3, 7]	4 [2, 6]	6 [3, 8]
History of fever, n (%)	87/87 (100.0)	67/67 (100.0)	20/20 (100.0)
Night sweats, n (%)	64/87 (73.6)	49/67 (73.1)	15/20 (75.0)
Headache, n (%)	69/87 (79.3)	53/67 (79.1)	16/20 (80.0)
Cough, n (%)	53/87 (60.9)	38/67 (56.7)	15/20 (75.0)
Diarrhea, n (%)	30/87 (34.5)	20/67 (29.9)	10/20 (50.0)
Shortness of breath, n (%)	19/87 (21.8)	11/67 (16.4)	8/20 (40.0)
Dysuria, n (%)	8/87 (9.2)	5/67 (7.5)	3/20 (15.0)
Received antibiotic or antimalarial agent prior to admission, n (%)	32/87 (36.8)	26/67 (38.8)	6/20 (30.0)
Temperature ≥38°C, n (%)	28/87 (32.2)	20/67 (29.9)	8/20 (40.0)
Temperature <36°C, n (%)	26/87 (29.9)	19/67 (28.4)	7/20 (35.0)
Heart rate, beats/min, median [IQR]	98 [90, 111]	98 [86, 111]	103 [98, 111]
Respiratory rate, beats/min, median [IQR]	22 [20, 26]	22 [20, 28]	22 [22, 24]
Systolic blood pressure, mmHg, median [IQR]	100 [92, 113]	102 [95, 118]	94 [90, 108]
Oxygen saturation, %, median [IQR]	97 [95, 98]	97 [96, 98]	97 [95, 98]
Encephalopathy, n (%)[a]	17/87 (19.5)	13/67 (19.4)	4/20 (20.0)
qSOFA score ≥2, n (%)[b]	41/87 (47.1)	28/67 (41.8)	13/20 (65.0)
qSOFA score ≥1, n (%)[b]	79/87 (90.8)	61/67 (91.0)	18/20 (90.0)
Modified SIRS score ≥2, n (%)[c]	74/87 (85.1)	55/67 (82.1)	19/20 (95.0)
MEWS, median [IQR]	3 [2, 5]	3 [2, 5]	5 [3, 5]
UVA score, median [IQR]	2 [2, 4]	2 [2, 4]	2 [2, 5]
Shock, n (%)[d]	13/87 (14.9)	7/67 (10.4)	6/20 (30.0)
Acute respiratory failure, n (%)[e]	22/87 (25.3)	17/67 (25.4)	5/20 (25.0)
Severe anemia, n (%)[f]	17/87 (19.5)	13/67 (19.4)	4/20 (20.0)
HIV-infected, n (%)	48/87 (55.2)	35/67 (52.2)	13/20 (65.0)
WHO clinical stage 3 or 4 , n (%)	34/48 (70.8)	25/35 (71.4)	9/13 (69.2)
Newly diagnosed HIV-infection, n (%)	8/48 (16.7)	7/35 (20.0)	1/13 (7.7)
On ART prior to admission, n (%)[g]	28/40 (70.0)	17/28 (60.7)	11/12 (91.7)
On TMP-SMX prior to admission, n (%)[g]	29/40 (72.5)	18/28 (64.3)	11/12 (91.7)
Malaria RDT positive, n (%)	21/86 (24.4)	13/66 (19.7)	8/20 (40.0)
Microbiological TB positive, n (%)[h]<br>Urine TB-LAM positive	16/87 (18.4)<br>13/39 (33.3)	8/67 (11.9)<br>7/30 (23.3)	8/20 (40.0)<br>6/9 (66.7)
Influenza PCR positive, n (%)	3/78 (3.8)	3/60 (5.0)	0/18 (0.0)
Death in-hospital or transfer, n (%)	12/87 (13.8)	6/67 (9.0)	6/20 (30.0)
Duration of hospitalization, days, median [IQR][i]	5 [3, 8]	5 [3, 8]	5 [3, 8]
KPS ≤70 at alive discharge, n (%)	8/73 (11.0)	4/60 (6.7)	4/13 (30.8)
Death at 30-days post-discharge, n (%)	18/81 (22.2)	10/62 (16.1)	8/19 (42.1)

Abbrevations: IQR: interquartile range, qSOFA: quick Sequential (Sepsis-related) Organ Failure Assessment, SIRS: Systemic Inflammatory Response Syndrome, MEWS: Modified Early Warning Score, UVA: universal vital assessment, HIV: human immunodeficiency virus, WHO: World Health Organization, ART: anti-retroviral therapy, RDT: rapid diagnostic test, TB: tuberculosis, LAM: lipoarabinomannan, PCR: polymerase chain reaction

Legend: [a]Anything other than “Alert” on AVPU (alert, responsive to voice, responsive to pain, unresponsive) mental status assessment; [b]Systolic blood pressure ≤100mmHg, respiratory rate ≥22

breaths/min, and encephalopathy, latter defined using AVPU scale; [c]Temperature ≥38°C or <36°C, heart rate ≥90 beats/min, respiratory rate ≥20 breaths/min; [d]Systolic blood pressure ≤90 mmHg despite administration of ≥1 liter of intravenous fluid; [e]Oxygen saturation ≤90% or respiratory rate ≥30 breaths/min; [f]Hemoglobin <9 g/dl or administration of blood transfusion. [g]Denominator is number with known HIV-infection prior to admission; [h]Sputum Xpert Ultra or smear or urine TB-LAM positive; [i]Unknown for 4 patients.

Table E4: Concentrations of soluble mediators in the discovery cohort stratified by immune subtype

Mediator	All patients (N=201)	Subtype 1 (N=105)	Subtype 2 (N=96)	p-value[a]
IFN-γ, log pg/ml, median [IQR]	0.15 [-0.23, 0.64]	-0.12 [-0.49, 0.23]	0.48 [0.12, 1.06]	<0.001
IL-6, log pg/ml, median [IQR]	1.20 [0.56, 1.67]	0.68 [0.17, 1.20]	1.60 [1.24, 2.04]	<0.001
IL-8, log pg/ml, median [IQR]	1.23 [0.87, 1.58]	1.04 [0.83, 1.35]	1.47 [1.07, 1.73]	<0.001
IL-10, log pg/ml, median [IQR]	1.16 [0.49, 1.74]	0.74 [-0.30, 1.21]	1.53 [1.10, 2.51]	<0.001
IP-10/CXCL10, log pg/ml, median [IQR]	2.89 [2.35, 4.00]	2.45 [2.04, 2.82]	3.91 [3.05, 4.74]	<0.001
MIP-1α/CCL3, log pg/ml, median [IQR]	1.45 [1.15, 1.79]	1.28 [1.00, 1.63]	1.58 [1.28, 1.96]	<0.001
MIP-1β/CCL4, log pg/ml, median [IQR]	1.79 [1.58, 1.98]	1.67 [1.52, 1.85]	1.92 [1.69, 2.07]	<0.001
TNF-α, log pg/ml, median [IQR]	1.83 [1.58, 2.05]	1.60 [1.40, 1.82]	2.05 [1.86, 2.27]	<0.001
Angiopoetin-1, log pg/ml, median [IQR]	4.56 [4.30, 4.74]	4.64 [4.46, 4.80]	4.42 [4.22, 4.62]	<0.001
Angiopoetin-2, log pg/ml, median [IQR]	3.51 [3.29, 3.80]	3.40 [3.18, 3.66]	3.64 [3.42, 3.99]	<0.001
sIL-2Ra, log pg/ml, median [IQR]	3.32 [3.00, 3.62]	3.05 [2.88, 3.29]	3.59 [3.39, 3.88]	<0.001
sTNFR1, log pg/ml, median [IQR]	3.44 [3.27, 3.70]	3.30 [3.15, 3.43]	3.67 [3.49, 3.86]	<0.001
MIF, log pg/ml, median [IQR]	2.90 [2.63, 3.25]	2.73 [2.48, 2.94]	3.18 [2.87, 3.50]	<0.001
PAI-1, log pg/ml, median [IQR]	4.99 [4.77, 5.15]	4.96 [4.74, 5.15]	5.01 [4.78, 5.17]	0.534
IFN-γ/IL-10, log pg/ml, median [IQR]	0.16 [-0.20, 0.50]	-0.01 [-0.44, 0.32]	0.28 [0.04, 0.62]	<0.001
IL-6/IL-10, log pg/ml, median [IQR]	0.61 [0.16, 1.37]	0.23 [-0.08, 1.07]	0.94 [0.53, 1.60]	<0.001
TNF-α/sTNFR1, log pg/ml, median [IQR]	0.53 [0.46, 0.58]	0.48 [0.42, 0.54]	0.55 [0.51, 0.61]	<0.001
Angiopoietin-2/Angiopoietin-1, log pg/ml, median [IQR]	0.78 [0.72, 0.86]	0.74 [0.67, 0.80]	0.85 [0.76, 0.91]	<0.001

Abbrevations: IQR: interquartile range, IFN: interferon, IL: interleukin, IP-10/CXCL10: IFN-γ-induced protein-10/C-X-C motif chemokine 10, MIP-1α/CCL3: macrophage inflammatory protein-1-alpha/chemokine (C-C motif) ligand 3, MIP-1β/CCL4: macrophage inflammatory protein-1-beta/chemokine (C-C motif) ligand 4, TNF-α: tumor necrosis factor-α, sIL-2RA/sCD25: soluble IL-2 receptor alpha/soluble CD25, sTNFR1: soluble TNF-receptor type 1, MIF: macrophage migration inhibitory factor, PAI-1: plasminogen activator inhibitor-1.

Legend: [a]Wilcoxon rank-sum test

Table E5: Concentrations of soluble mediators in the validation cohort stratified by immune subtype

Mediator	All patients (N=87)	Subtype 1 (N=67)	Subtype 2 (N=20)	p-value[a]
IFN-γ, log pg/ml, median [IQR]	0.15 [-0.28, 0.68]	0.03 [-0.40, 0.32]	0.86 [0.46, 1.55]	<0.001
IL-6, log pg/ml, median [IQR]	0.98 [0.55, 1.61]	0.77 [0.53, 1.20]	1.65 [1.45, 2.06]	<0.001
IL-8, log pg/ml, median [IQR]	1.08 [0.81, 1.48]	1.02 [0.81, 1.32]	1.54 [1.20, 2.00]	0.001
IL-10, log pg/ml, median [IQR]	1.15 [0.54, 1.85]	0.93 [0.42, 1.48]	2.19 [1.03, 2.68]	0.002
IP-10/CXCL10, log pg/ml, median [IQR]	2.85 [2.37, 3.93]	2.66 [2.24, 3.25]	4.74 [3.15, 4.80]	<0.001
MIP-1α/CCL3, log pg/ml, median [IQR]	1.40 [1.09, 1.66]	1.35 [1.01, 1.60]	1.55 [1.34, 1.77]	0.058
MIP-1β/CCL4, log pg/ml, median [IQR]	1.72 [1.53, 1.87]	1.65 [1.53, 1.83]	1.81 [1.53, 2.14]	0.039
TNF-α, log pg/ml, median [IQR]	1.72 [1.54, 1.96]	1.67 [1.47, 1.83]	2.15 [1.93, 2.37]	<0.001
Angiopoetin-1, log pg/ml, median [IQR]	4.53 [4.22, 4.70]	4.61 [4.38, 4.71]	4.31 [3.48, 4.47]	0.001
Angiopoetin-2, log pg/ml, median [IQR]	3.52 [3.28, 3.81]	3.45 [3.19, 3.69]	3.77 [3.50, 4.26]	0.001
sIL-2Ra, log pg/ml, median [IQR]	3.31 [3.04, 3.67]	3.22 [2.94, 3.40]	3.82 [3.67, 4.11]	<0.001
sTNFR1, log pg/ml, median [IQR]	3.49 [3.28, 3.65]	3.41 [3.20, 3.54]	3.72 [3.59, 4.09]	<0.001
MIF, log pg/ml, median [IQR]	2.92 [2.53, 3.17]	2.81 [2.49, 3.12]	3.15 [2.82, 3.51]	0.002
PAI-1, log pg/ml, median [IQR]	4.96 [4.76, 5.14]	4.91 [4.76, 5.12]	5.02 [4.78, 5.21]	0.308
IFN-γ/IL-10, log pg/ml, median [IQR]	0.16 [-0.16, 0.52]	0.12 [-0.28, 0.38]	0.54 [0.10, 0.98]	0.006
IL-6/IL-10, log pg/ml, median [IQR]	0.60 [0.29, 1.35]	0.59 [0.24, 1.35]	0.72 [0.53, 1.34]	0.250
TNF-α/sTNFR1, log pg/ml, median [IQR]	0.50 [0.43, 0.57]	0.50 [0.43, 0.54]	0.57 [0.48, 0.66]	0.007
Angiopoietin-2/Angiopoieitin-1, log pg/ml, median [IQR]	0.79 [0.73, 0.88]	0.77 [0.70, 0.84]	0.87 [0.80, 1.19]	<0.001

Abbrevations: IQR: interquartile range, IFN: interferon, IL: interleukin, IP-10/CXCL10: IFN-γ-induced protein-10/C-X-C motif chemokine 10, MIP-1α/CCL3: macrophage inflammatory protein-1-alpha/chemokine (C-C motif) ligand 3, MIP-1β/CCL4: macrophage inflammatory protein-1-beta/chemokine (C-C motif) ligand 4, TNF-α: tumor necrosis factor-α, sIL-2RA/sCD25: soluble IL-2 receptor alpha/soluble CD25, sTNFR1: soluble TNF-receptor type 1, MIF: macrophage migration inhibitory factor, PAI-1: plasminogen activator inhibitor-1.

Legend: [a]Wilcoxon rank-sum test

Table E6

Table E6A: Association between immune mediator subtype (2 vs. 1) and 30-day mortality, unadjusted and adjusted for age, sex, duration of illness, and Modified Early Warning Score in pooled population of discovery and validation cohorts (N=259)

Variable	Univariable OR (95% CI)	p-value	Multivariable OR (95% CI)	p-value
Death at 30-days post-discharge, n (%)	2.71 (1.51-4.86)	0.001	2.34 (1.24-4.41)	0.008

Table E6B: Association between immune mediator subtype (2 vs. 1) and 30-day mortality, unadjusted and adjusted for age, sex, duration of illness, and Universal Vital Assessment score in pooled population of discovery and validation cohorts (N=259)

Variable	Univariable OR (95% CI)	p-value	Multivariable OR (95% CI)	p-value
Death at 30-days post-discharge, n (%)	2.71 (1.51-4.86)	0.001	2.59 (1.36-4.92)	0.004

Table E6C: Association between immune mediator subtype (2 vs. 1) and 30-day mortality, unadjusted and adjusted for age, sex, duration of illness, and quick Sepsis-related Organ Failure Assessment score in pooled population of discovery and validation cohorts (N=259)

Variable	Univariable OR (95% CI)	p-value	Multivariable OR (95% CI)	p-value
Death at 30-days post-discharge, n (%)	2.71 (1.51-4.86)	0.001	2.43 (1.29-4.56)	0.006

Abbreviations: OR: odds ratio; CI: confidence interval

Table E7: Patient characteristics stratified by transcriptional subtype

Patient characteristic	All patients (N=128)	Transcriptional Subtype 1 (N=100)	Transcriptional Subtype 2 (N=28)	p-value[a]
Female sex, n (%)	80/128 (62.5)	62/100 (62.0)	18/28 (64.3)	1.000
Age, years, median [IQR]	33 [26, 43]	33 [26, 43]	32 [27, 39]	0.831
Duration of illness prior to admission, days, median [IQR][b]	4 [3, 7]	4 [3, 7]	6 [3, 7]	0.204
History of fever, n (%)	128/128 (100.0)	100/100 (100.0)	28/28 (100.0)	--
Night sweats, n (%)	99/128 (77.3)	77/100 (77.0)	22/28 (78.6)	1.000
Headache, n (%)	104/128 (81.2)	85/100 (85.0)	19/28 (67.9)	0.075
Cough, n (%)	72/128 (56.2)	53/100 (53.0)	19/28 (67.9)	0.236
Diarrhea, n (%)	48/128 (37.5)	31/100 (31.0)	17/28 (60.7)	0.008
Shortness of breath, n (%)	29/128 (22.7)	19/100 (19.0)	10/28 (35.7)	0.107
Dysuria, n (%)	15/128 (11.7)	10/100 (10.0)	5/28 (17.9)	0.418
Received antibiotic or antimalarial agent prior to admission, n (%)	45/128 (35.2)	33/100 (33.0)	12/28 (42.9)	0.458
Temperature ≥38°C, n (%)	47/128 (36.7)	37/100 (37.0)	10/28 (35.7)	1.000
Temperature <36°C, n (%)	44/128 (34.4)	34/100 (34.0)	10/28 (35.7)	1.000
Heart rate, beats/min, median [IQR]	97 [85, 110]	96 [84, 107]	103 [93, 114]	0.103
Respiratory rate, beats/min, median [IQR]	22 [22, 27]	22 [20, 26]	25 [22, 29]	0.005
Systolic blood pressure, mmHg, median [IQR]	106 [95, 118]	107 [98, 118]	99 [90, 110]	0.122
Oxygen saturation, %, median [IQR]	97 [96, 98]	97 [96, 98]	98 [95, 98]	0.581
Encephalopathy, n (%)[c]	19/128 (14.8)	12/100 (12.0)	7/28 (25.0)	0.159
qSOFA score ≥2, n (%)[d]	53/128 (41.4)	34/100 (34.0)	19/29 (67.9)	0.003
qSOFA score ≥1, n (%)[d]	112/128 (87.5)	85/100 (85.0)	27 (96.4)	0.196
Modified SIRS score ≥2, n (%)[e]	111/128 (86.7)	87/100 (87.0)	24 (85.7)	1.000
MEWS, median [IQR]	3 [2, 4]	3 [2, 4]	4 [3, 6]	0.001
UVA score, median [IQR]	3 [2, 4]	2 [1, 4]	4 [2, 5]	0.013
Shock, n (%)[f]	17/128 (13.3)	11/100 (11.0)	6/28 (21.4)	0.262
Acute respiratory failure, n (%)[g]	30/128 (23.4)	20/100 (20.0)	10/29 (35.7)	0.138
Severe anemia, n (%)[h]	29/128 (22.7)	21/100 (21.0)	8/28 (28.6)	0.555
HIV-infected, n (%)	69/127 (53.9)	48/99 (48.5)	21/28 (75.0)	0.013
WHO clinical stage 3 or 4, n (%)	51/69 (73.9)	32/48 (66.7)	19/21 (90.5)	0.043
Newly diagnosed HIV infection, n (%)	13/69 (18.8)	11/48 (22.9)	2/21 (9.5)	0.317
On ART prior to admission, n (%)[i]	38/56 (67.9)	26/37 (70.3)	12/19 (63.2)	0.589
On TMP-SMX prior to admission, n (%)[i]	43/56 (76.8)	30/37 (81.1)	13/19 (68.4)	0.288
Malaria RDT positive, n (%)	27/124 (21.8)	24/98 (24.5)	3/26 (11.5)	0.190
Microbiological TB positive, n (%)[j]	20/128 (15.6)	10/100 (10.0)	10/28 (35.7)	0.003
Urine TB-LAM positive	20/55 (36.4)	10/37 (27.0)	10/18 (55.6)	0.039
Influenza PCR positive, n (%)	2/108 (1.9)	2/85 (2.4)	0/23 (0.0)	1.000
Death in-hospital or transfer, n (%)	19/108 (14.8)	15/100 (15.0)	4/28 (14.3)	1.000
Duration of hospitalization, days, median [IQR][k]	5 [4, 7]	4 [3, 7]	7 [4, 9]	0.010
KPS ≤70 at alive discharge, n (%)	9/108 (8.3)	4/84 (4.8)	5/24 (20.8)	0.036
Death at 30-days post-discharge, n (%)	30/117 (25.6)	19/92 (20.7)	11/25 (44.0)	0.035

Abbrevations: IQR: interquartile range, qSOFA: quick Sequential (Sepsis-related) Organ Failure Assessment, SIRS: Systemic Inflammatory Response Syndrome, MEWS: Modified Early Warning Score, UVA: universal vital assessment, HIV: human immunodeficiency virus, WHO: World Health Organization, ART: anti-retroviral therapy, RDT: rapid diagnostic test, TB: tuberculosis, LAM: lipoarabinomannan, PCR: polymerase chain reaction; KPS: Karnofsky performance status.

Legend: [a]Fisher Exact or Chi-squared test with continuity correction; [b]Unknown for 1 patient; [c]Anything other than "Alert" on AVPU (alert, responsive to voice, responsive to pain, unresponsive) mental status assessment; [d]Systolic blood pressure ≤100mmHg, respiratory rate ≥22 breaths/min, and encephalopathy, latter defined using AVPU scale; [e]Temperature ≥38°C or <36°C, heart rate ≥90 beats/min, respiratory rate ≥20 breaths/min; [f]Systolic blood pressure ≤90 mmHg despite administration of ≥1 liter of intravenous fluid; [g]Oxygen saturation ≤90% or respiratory rate ≥30 breaths/min; [h]Hemoglobin <9 g/dl or administration of blood transfusion. [i]Denominator is number with known HIV-infection prior to admission; [j]Sputum Xpert Ultra or smear or urine TB-LAM positive; [k]Unknown for 9 patients.

Table E8

Table E8A: Association between transcriptional subtype (2 vs. 1) and 30-day mortality, unadjusted and adjusted for age, sex, duration of illness, and Modified Early Warning Score (N=116)

Variable	Univariable OR (95% CI)	p-value	Multivariable OR (95% CI)	p-value
Death at 30-days post-discharge, n (%)	3.02 (1.18-7.71)	0.021	2.39 (0.81-7.06)	0.115

Table E8B: Association between transcriptional subtype (2 vs. 1) and 30-day mortality, unadjusted and adjusted for age, sex, duration of illness, and Universal Vital Assessment score (N=116)

Variable	Univariable OR (95% CI)	p-value	Multivariable OR (95% CI)	p-value
Death at 30-days post-discharge, n (%)	3.02 (1.18-7.71)	0.021	2.54 (0.89-7.28)	0.083

Table E8C: Association between transcriptional subtype (2 vs. 1) and 30-day mortality, unadjusted and adjusted for age, sex, duration of illness, and quick Sepsis-related Organ Failure Assessment score (N=116)

Variable	Univariable OR (95% CI)	p-value	Multivariable OR (95% CI)	p-value
Death at 30-days post-discharge, n (%)	3.02 (1.18-7.71)	0.021	2.35 (0.81-6.84)	0.115

Abbreviations: OR: odds ratio; CI: confidence interval

Table E9: Characteristics of patients assigned to immunologically similar and dissimilar host subtypes

Patient characteristic	All patients (N=122)	Assigned to dissimilar subtype (N=47)	Assigned to similar subtype (N=75)	P-value[a]
Female sex, n (%)	78/122 (63.9)	28/47 (59.6)	50/75 (66.7)	0.548
Age, years, median [IQR]	33 [26,43]	30 [26,40]	35 [27,44]	0.177
Duration of illness prior to admission, days, median [IQR][b]	4 [3,7]	5 [3,7]	4 [2,6]	0.204
qSOFA score ≥2, n (%)[c]	50/122 (41)	18/47 (38.3)	32/75 (42.7)	0.773
qSOFA score ≥1, n (%)[c]	108/122 (88.5)	41/47 (87.2)	67/75 (89.3)	0.950
Modified SIRS score ≥2, n (%)[d]	107/122 (87.7)	42/47 (89.4)	65/75 (86.7)	0.781
MEWS, median [IQR]	3 [2,4]	3 [2,5]	3 [2,4]	0.323
UVA score, median [IQR]	3 [2,4]	2 [2,4]	3 [2,4]	0.249
Shock, n (%)[e]	17/122 (13.9)	6/47 (12.8)	11/75 (14.7)	0.979
Acute respiratory failure, n (%)[f]	28/122 (23.0)	10/47 (21.3)	18/75 (24.0)	0.899
Severe anemia, n (%)[g]	26/122 (21.3)	10/47 (21.3)	16/75 (21.3)	1.000
HIV-infected, n (%)	67/121 (55.3)	28/47 (59.6)	39/74 (52.7)	0.459
Malaria RDT positive, n (%)	26/118 (22.0)	14/45 (31.1)	12/73 (16.4)	0.062
Microbiological TB positive, n (%)[h]	20/122 (16.4)	7/47 (14.9)	13/75 (17.3)	0.918
Influenza PCR positive, n (%)	2/104 (1.9)	1/39 (2.6)	1/65 (1.5)	1.000
Death in-hospital or transfer, n (%)	17/122 (13.9)	7/47 (14.9)	10/75 (13.3)	1.000
Duration of hospitalization, days, median [IQR][i]	5 [3,7]	5 [3,8]	5 [4,7]	0.687
KPS ≤ 70 at alive discharge, n (%)	9/104 (8.7)	5/39 (12.8)	4/65 (6.2)	0.290
Death at 30-days post-discharge, n (%)	29/112 (25.9)	13/43 (30.2)	16/69 (23.2)	0.408

Abbrevations: IQR: interquartile range, qSOFA: quick Sequential (Sepsis-related) Organ Failure Assessment, SIRS: Systemic Inflammatory Response Syndrome, MEWS: Modified Early Warning Score, UVA: universal vital assessment, HIV: human immunodeficiency virus, RDT: rapid diagnostic test, TB: tuberculosis, PCR: polymerase chain reaction

Legend: [a]Fisher exact, Chi-square, or Wilcoxon rank-sum test, [b]Unknown for 1 patient, [c]Systolic blood pressure ≤100mmHg, respiratory rate ≥22 breaths/min, and encephalopathy, latter defined using alert, responsive to voice, responsive to pain, unresponsive (AVPU) mental status assessment; [d]Temperature ≥38°C or <36°C, heart rate ≥90 beats/min, respiratory rate ≥20 breaths/min; [e]Systolic blood pressure ≤90 mmHg despite administration of ≥1 liter of intravenous fluid; [f]Oxygen saturation ≤90% or respiratory rate ≥30 breaths/min; [g]Hemoglobin <9 g/dl or administration of blood transfusion; [h]Sputum Xpert Ultra or smear or urine TB-LAM positive; [i]Unknown for 9 patients.

Supplemental Figure Legends

Figure E1: Overview of immune mediator and transcriptional subtyping approach. Of the 288 patients for whom serum mediators were quantified, patients were randomly split into discovery (70%; N=201) and internal validation (30%; N=87) sub-cohorts using the caTools R package.

Figure E2: Study flow diagram.

Figure E3: Immune mediator-derived clusters (subtypes) in internal validation cohort. (A) Unsupervised hierarchical clustering of 14 serum mediators reflecting innate and adaptive immune activation, endothelial dysfunction, and fibrinolysis; dendrogram indicates cluster partition prior to k-means consolidation (N=87). **(B)** Optimal cluster partitions suggested by cluster stability and validation indices as per *NbClust* package. **(C)** First two principal components plotted with the proportion of variance explained by each component; individuals stratified by cluster (subtype) assignment (N=87). **(D)** Heatmap of z-score standardized serum mediator concentrations, stratified by cluster (subtype) assignment (N=87).

Figure E4: Concentrations of soluble mediators stratified by immune subtype in the discovery cohort (N=201)

Figure E5: Concentrations of soluble mediators stratified by immune subtype in the validation cohort (N=87)

Figure E6: Serum mediator concentrations over the reported course of illness in the discovery cohort, stratified by immune subtype. Concentrations of soluble mediators over the reported course of illness, with robust regression lines and 95% confidence intervals, stratified by immune subtype. (N=198 for **[A-E, G, H]**; N=196 for **[F]** as two patients with extreme outlier concentrations of Ang-1 excluded). For example, an individual data point corresponding to “day 0” represents the serum mediator concentration for a patient who was

admitted to hospital on the day of illness onset, while that corresponding to “day 5” represents a patient who was admitted to hospital on day 5 of illness.

Figure E7: Unsupervised hierarchical clustering and differential gene expression of whole-blood RNA-sequencing data. (**A**) Unsupervised hierarchical clustering of whole-blood RNA-sequencing data; dendrogram indicates cluster partition prior to k-means consolidation (N=128). (**B**) Volcano plot indicating differentially expressed genes across transcriptional clusters (subtypes) (N=128). Red shading indicates those genes differentially expressed at log-fold change ≥|1| and Benjamini-Hochberg adjusted p-value ≤0.01.

Figure E8: Alluvial diagrams demonstrating assignment of patients to soluble mediator and transcriptional subtypes (A; N=122) including those with and without HIV-associated TB (B; N=67) and malaria (C; N=118). Red shading in Panel A indicates proportion of patients assigned to immunologically dissimilar subtypes (i.e. soluble mediator subtype 2 and transcriptional subtype 1), grey shading indicates proportion of patients assigned to similar subtypes (i.e. soluble mediator subtype 2 and transcriptional subtype 2). Green shading in Panel B indicates proportion of patients with HIV-associated TB, grey shading indicates proportion of HIV-infected patients without TB. Blue shading in Panel C indicates proportion of patients with malaria, grey shading indicates proportion of patients without malaria.

Supplemental Figures

Figure E1

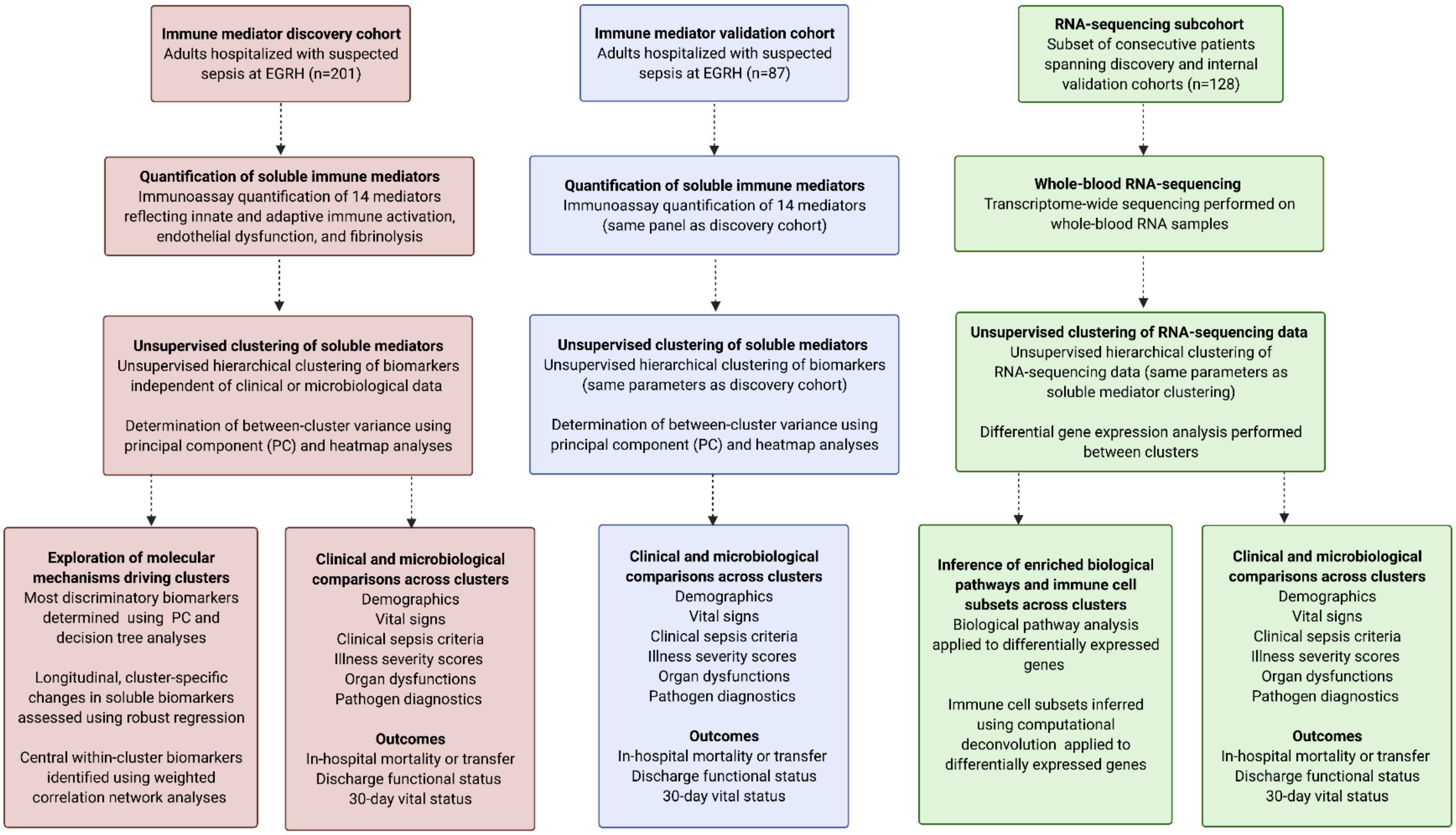

Figure E2

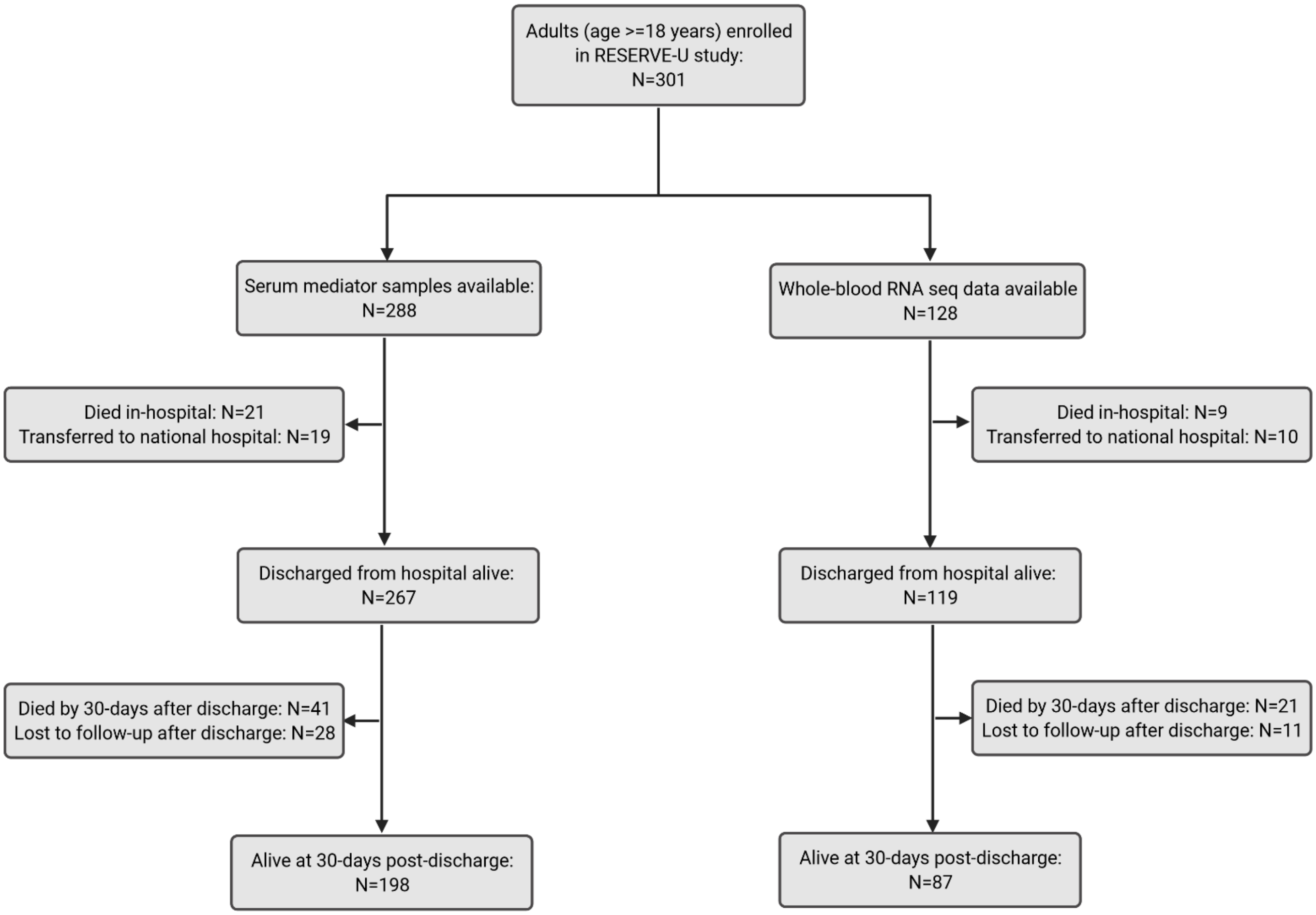
Adults (age >=18 years) enrolled in RESERVE-U study: N=301
Serum mediator samples available: N=288
Whole-blood RNA seq data available N=128
Died in-hospital: N=21
Transferred to national hospital: N=19
Died in-hospital: N=9
Transferred to national hospital: N=10
Discharged from hospital alive: N=267
Discharged from hospital alive: N=119
Died by 30-days after discharge: N=41
Lost to follow-up after discharge: N=28
Died by 30-days after discharge: N=21
Lost to follow-up after discharge: N=11
Alive at 30-days post-discharge: N=198
Alive at 30-days post-discharge: N=87

Figure E3

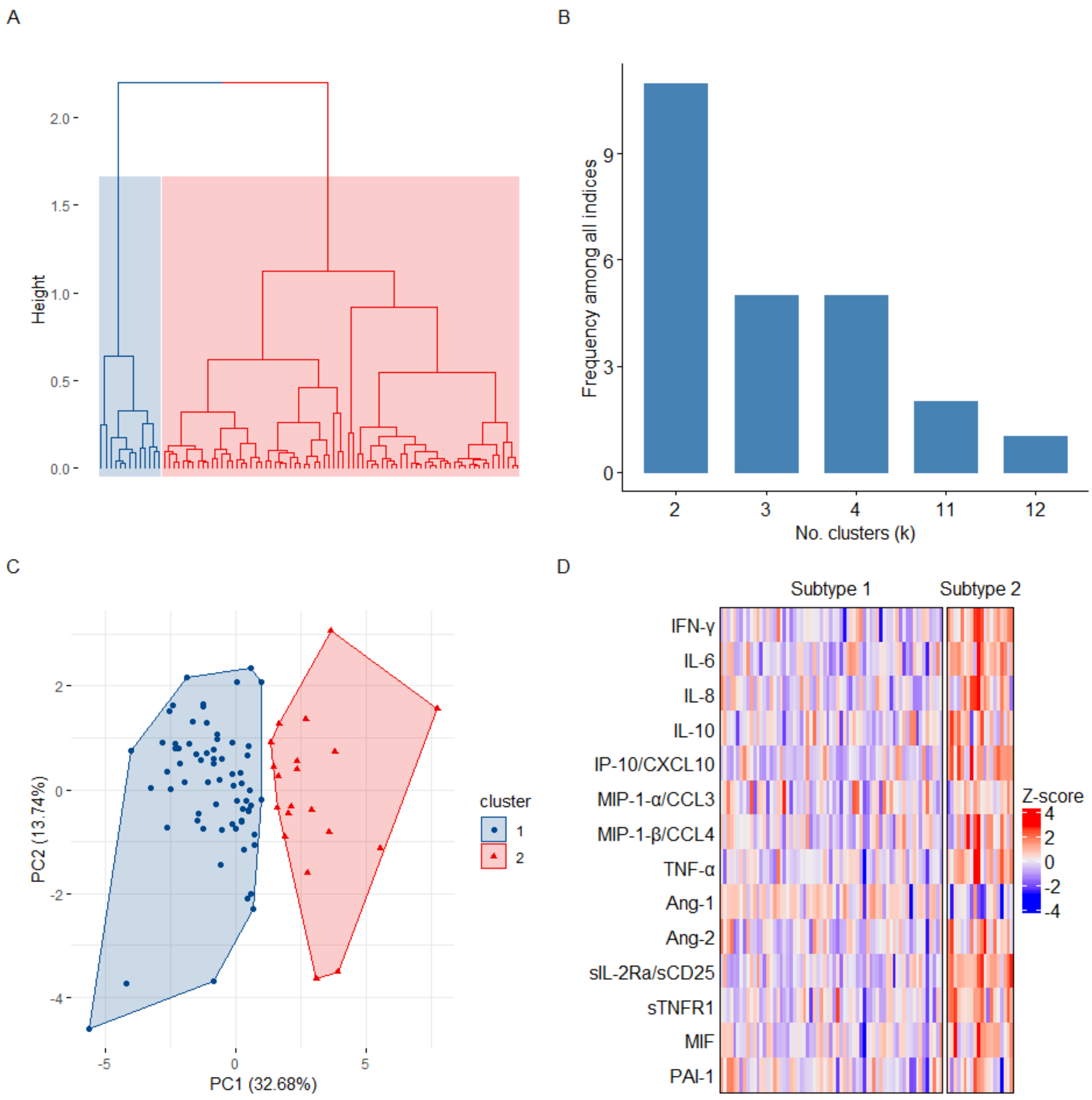
A
Height
2.0
1.5
1.0
0.5
0.0
B
Frequency among all indices
9
6
3
0
2
3
4
11
12
No. clusters (k)
C
PC2 (13.74%)
2
0
-2
-4
-5
0
5
PC1 (32.68%)
cluster
1
2
D
Subtype 1
Subtype 2
IFN-γ
IL-6
IL-8
IL-10
IP-10/CXCL10
MIP-1-α/CCL3
MIP-1-β/CCL4
TNF-α
Ang-1
Ang-2
sIL-2Ra/sCD25
sTNFR1
MIF
PAI-1
Z-score
4
2
0
-2
-4

Figure E4

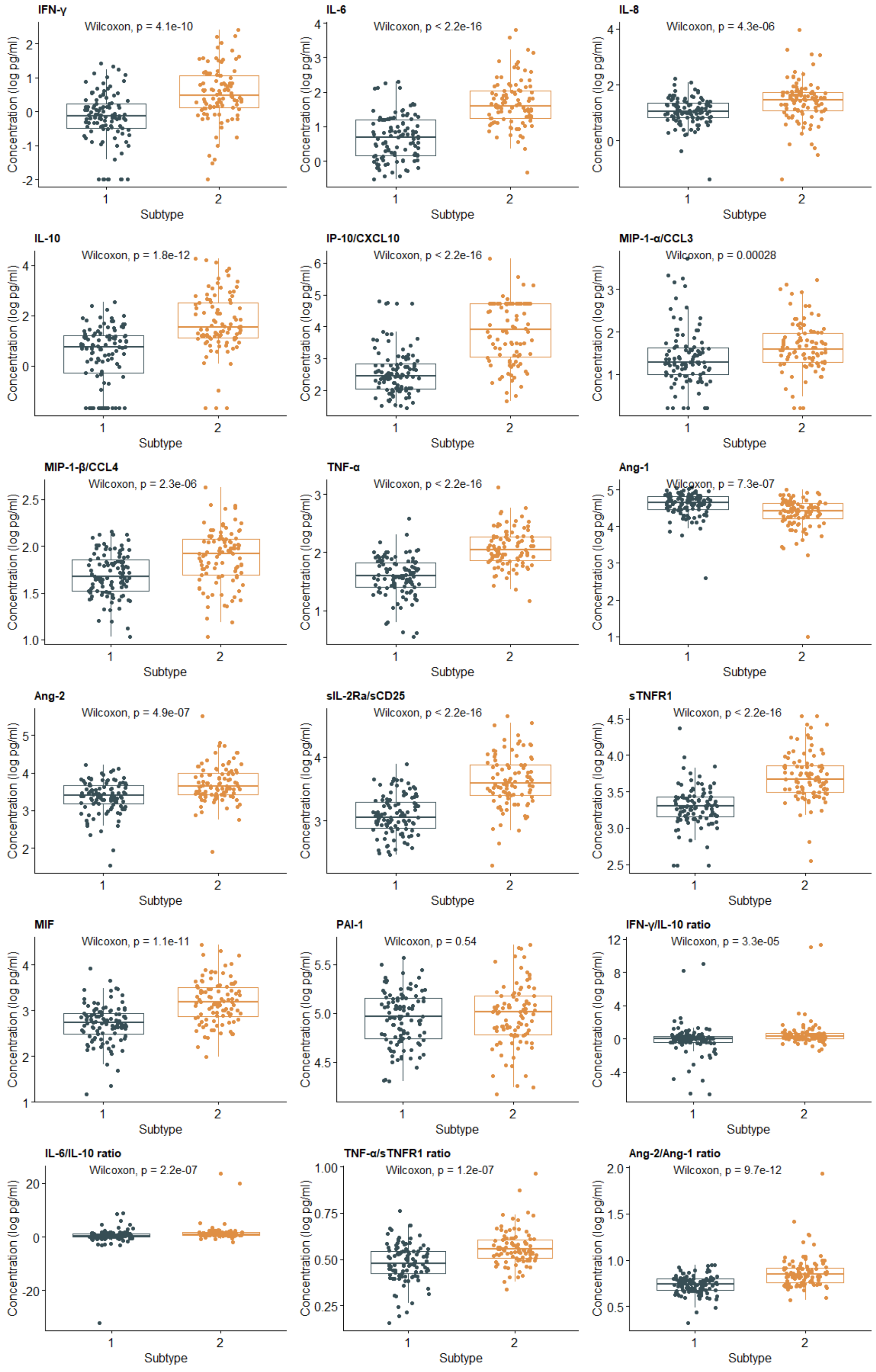
IFN-γ
Wilcoxon, p = 4.1e-10
IL-6
Wilcoxon, p < 2.2e-16
IL-8
Wilcoxon, p = 4.3e-06
IL-10
Wilcoxon, p = 1.8e-12
IP-10/CXCL10
Wilcoxon, p < 2.2e-16
MIP-1-α/CCL3
Wilcoxon, p = 0.00028
MIP-1-β/CCL4
Wilcoxon, p = 2.3e-06
TNF-α
Wilcoxon, p < 2.2e-16
Ang-1
Wilcoxon, p = 7.3e-07
Ang-2
Wilcoxon, p = 4.9e-07
sIL-2Ra/sCD25
Wilcoxon, p < 2.2e-16
sTNFR1
Wilcoxon, p < 2.2e-16
MIF
Wilcoxon, p = 1.1e-11
PAI-1
Wilcoxon, p = 0.54
IFN-γ/IL-10 ratio
Wilcoxon, p = 3.3e-05
IL-6/IL-10 ratio
Wilcoxon, p = 2.2e-07
TNF-α/sTNFR1 ratio
Wilcoxon, p = 1.2e-07
Ang-2/Ang-1 ratio
Wilcoxon, p = 9.7e-12
Concentration (log pg/ml)
Subtype
1
2

Figure E5

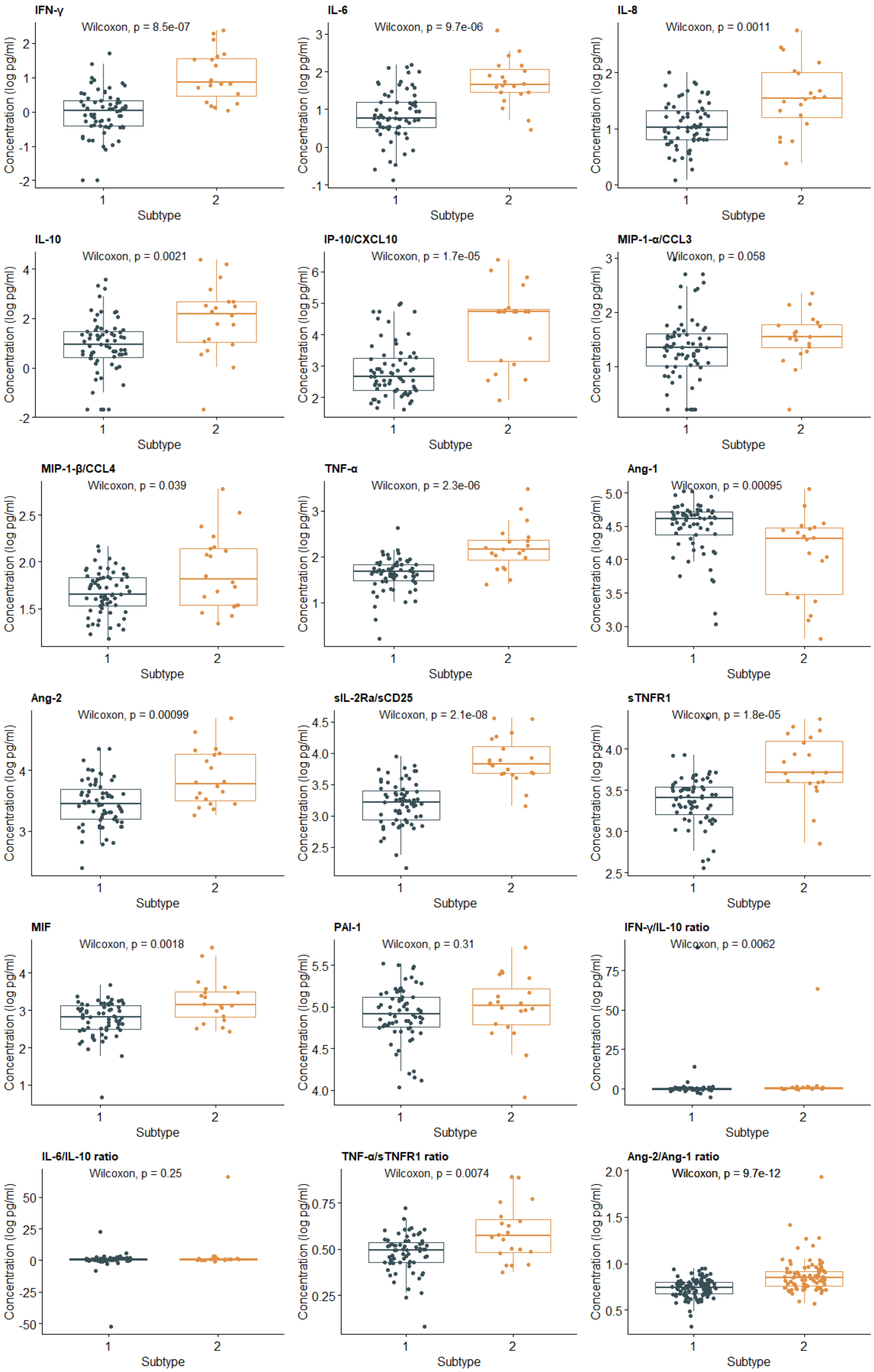
IFN-γ
Wilcoxon, p = 8.5e-07
IL-6
Wilcoxon, p = 9.7e-06
IL-8
Wilcoxon, p = 0.0011
IL-10
Wilcoxon, p = 0.0021
IP-10/CXCL10
Wilcoxon, p = 1.7e-05
MIP-1-α/CCL3
Wilcoxon, p = 0.058
MIP-1-β/CCL4
Wilcoxon, p = 0.039
TNF-α
Wilcoxon, p = 2.3e-06
Ang-1
Wilcoxon, p = 0.00095
Ang-2
Wilcoxon, p = 0.00099
sIL-2Ra/sCD25
Wilcoxon, p = 2.1e-08
sTNFR1
Wilcoxon, p = 1.8e-05
MIF
Wilcoxon, p = 0.0018
PAI-1
Wilcoxon, p = 0.31
IFN-γ/IL-10 ratio
Wilcoxon, p = 0.0062
IL-6/IL-10 ratio
Wilcoxon, p = 0.25
TNF-α/sTNFR1 ratio
Wilcoxon, p = 0.0074
Ang-2/Ang-1 ratio
Wilcoxon, p = 9.7e-12
Concentration (log pg/ml)
Subtype
1
2

Figure E6

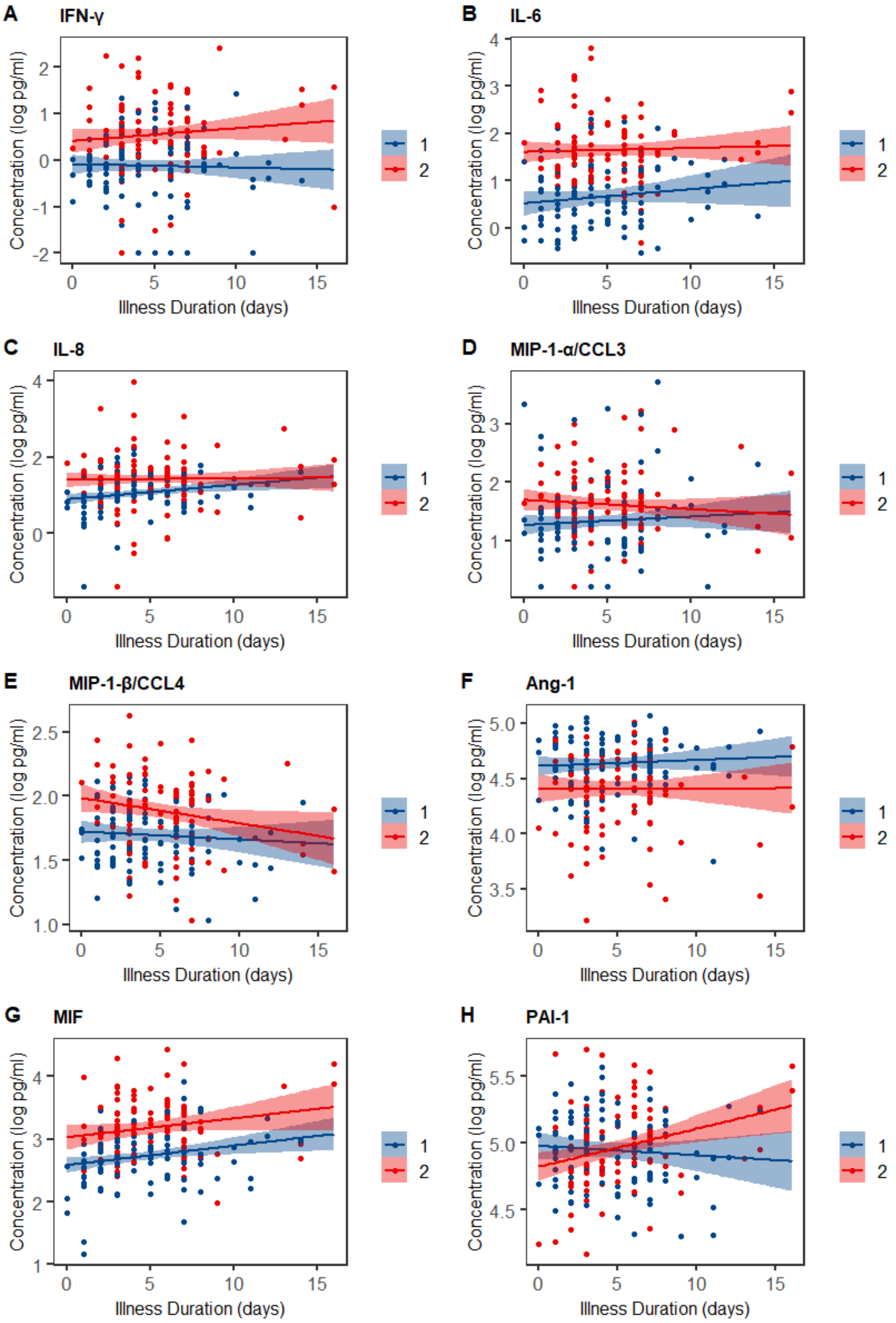
A
IFN-γ
B
IL-6
C
IL-8
D
MIP-1-α/CCL3
E
MIP-1-β/CCL4
F
Ang-1
G
MIF
H
PAI-1
Concentration (log pg/ml)
Illness Duration (days)
1
2

Figure E7

A

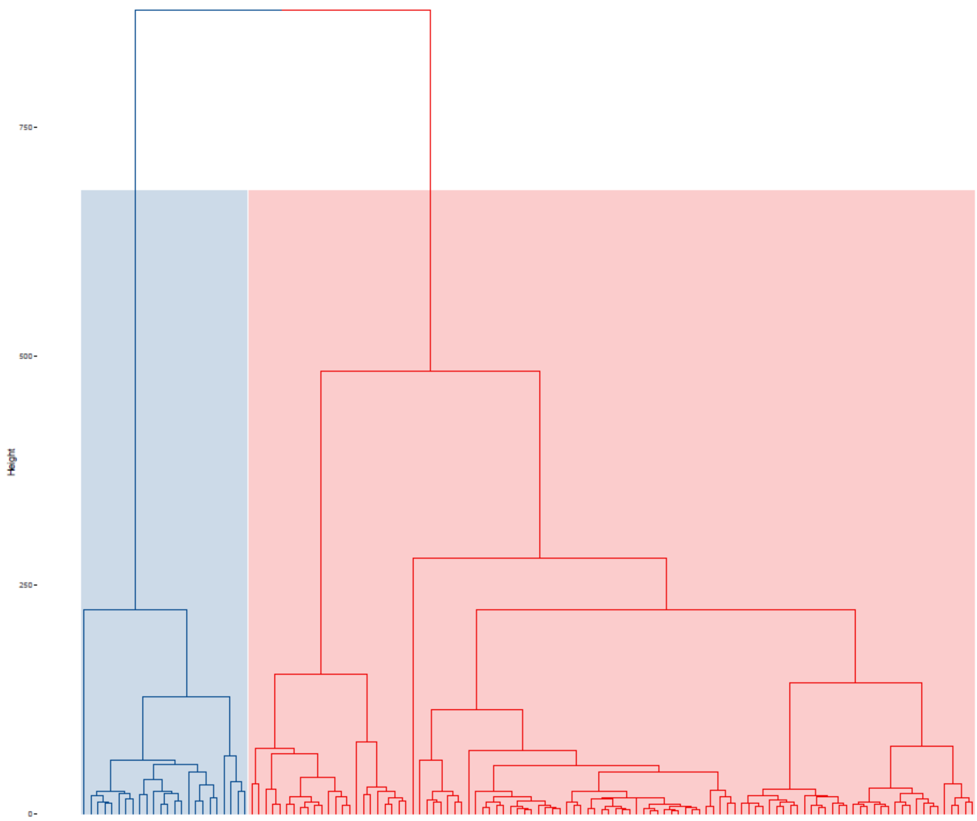
750
500
250
0
Height

B

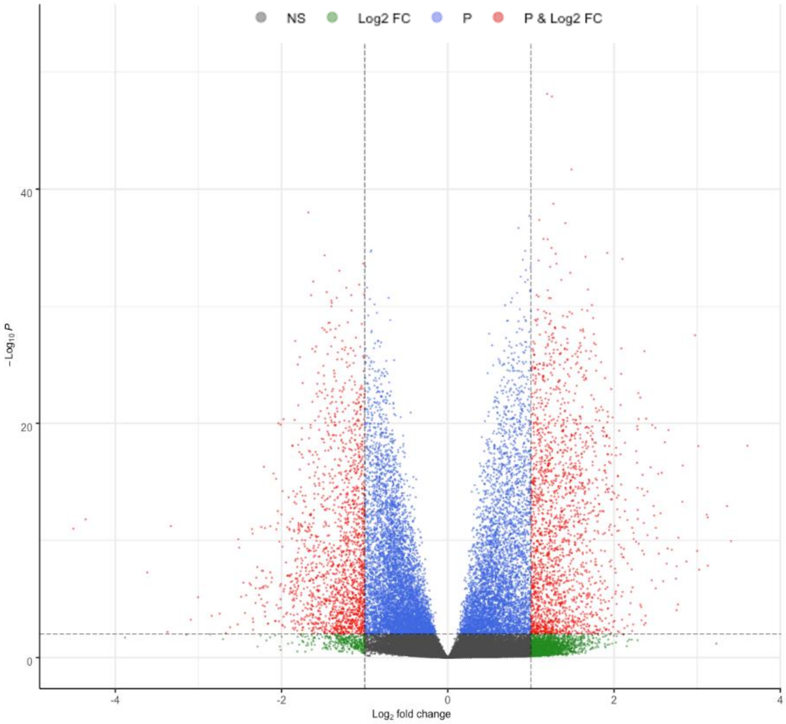
NS
Log2 FC
P
P & Log2 FC
40
20
0
$-\text{Log}_{10}\,P$
-4
-2
0
2
4
Log_2 fold change

Figure E8

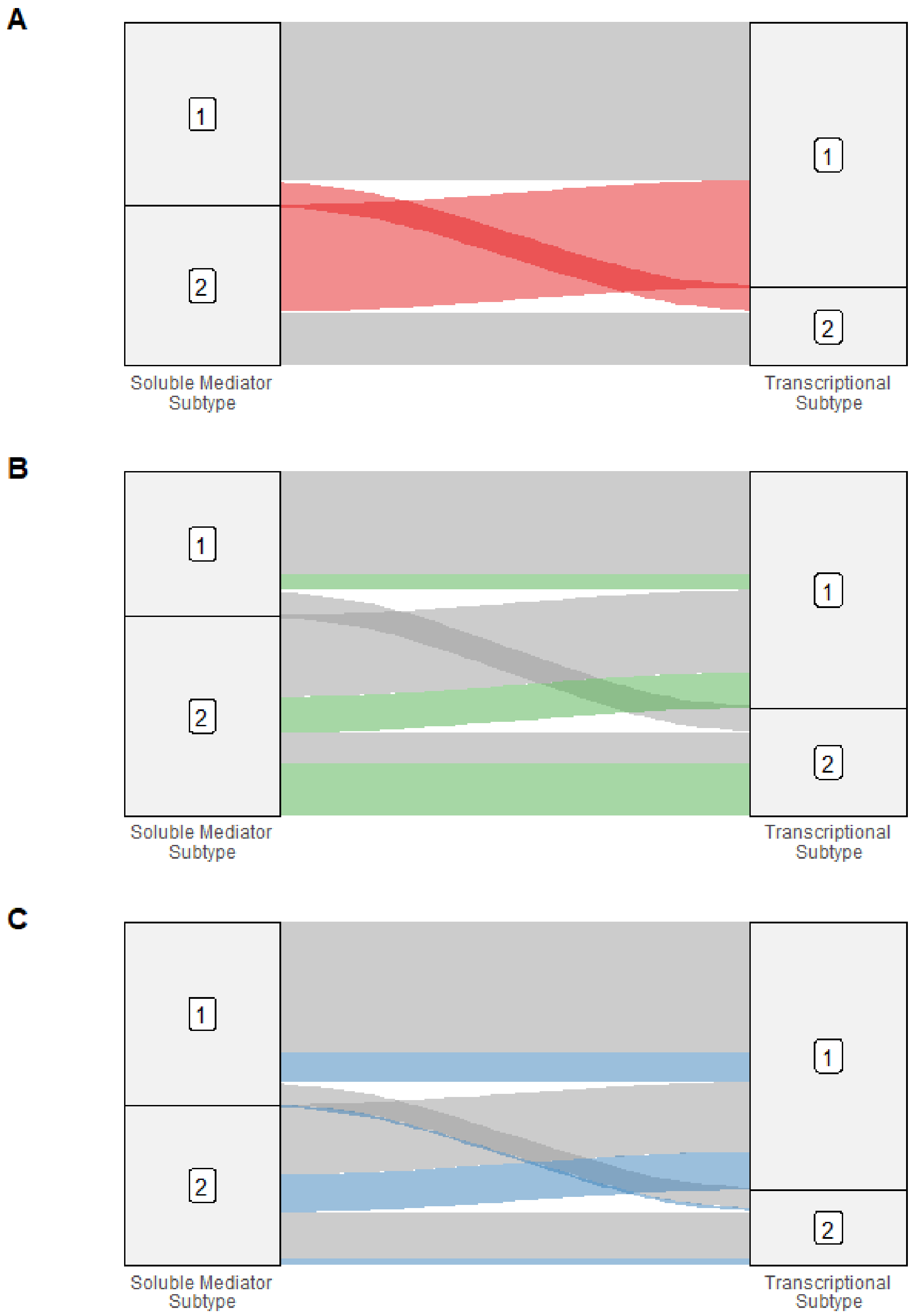
A
1
2
1
2
Soluble Mediator Subtype
Transcriptional Subtype
B
1
2
1
2
Soluble Mediator Subtype
Transcriptional Subtype
C
1
2
1
2
Soluble Mediator Subtype
Transcriptional Subtype
